# Supplementary material for: Phonemic composition influences words’ aesthetic appeal and memorability
Source: PLoS One. 2025 Dec 3;20(12):e0336597. doi: 10.1371/journal.pone.0336597 (PMC12674511; doi:10.1371/journal.pone.0336597)
Supplement: S1 Supporting information — (DOCX) [file pone.0336597.s001.docx]

# **S1 Supporting information: Appendix**

# **Phonemic composition influences words’ aesthetic appeal and memorability**

**Theresa Matzinger^# 1,2 *^ & David Košić^# 1^**

# joint first authors

1 Department of English and American Studies, University of Vienna, Vienna, Austria

2 Vienna Cognitive Science Hub, University of Vienna, Vienna, Austria

* corresponding author: [theresa.matzinger@univie.ac.at](mailto:theresa.matzinger@univie.ac.at)

# Exploratory analyses: the influence of phonotactic probability on aesthetic appeal and recall

In an exploratory study we investigated if phonotactic probabilities of the pseudowords have influenced either the appeal ratings or the recall performance, cf. [[1–3]](https://www.zotero.org/google-docs/?x4rw02).

## 1.1. Methods

We used the “Phonotactic Probability Calculator” by Vitevitch & Luce [[4]](https://www.zotero.org/google-docs/?qrj2NF) to extract two measures for each pseudoword: (1) *positional segment frequency* and (2) *biphone frequency*. *Positional segment frequency* was calculated as the sum of the positional frequencies of all individual phonemes in a word, while *biphone frequency* was calculated as the sum of the co-occurrence probabilities of all adjacent phoneme pairs (2-grams) in a word. These values were subsequently used in correlation analyses and entered as additional fixed effects in our models of perceived appeal and recall.

## 1.2. Results

### 1.2.1. Aesthetic appeal

The mean segment frequencies were highest in the neutral condition (0.41 ± SD 0.04), followed by the appealing (0.38 ± 0.01) and unappealing (0.29 ± 0.05) conditions. A similar pattern was observed for biphone frequencies (neutral: 0.024 ± 0.006; appealing: 0.018 ± 0.004; unappealing: 0.013 ± 0.006). Thus, overall, words with higher segment and biphone frequencies tended to receive higher ratings, but this relation did not hold consistently at the individual-word level (see Fig. 2 and Fig. A1). Statistical analyses confirmed that these associations were not significant: Spearman rank correlation coefficients showed that neither positional segment frequency nor biphone frequency significantly correlated with ratings of appeal, even though both showed non-significant trends towards medium positive relationships (positional segment frequency: rho = 0.580, p = 0.052; biphone frequency: rho = 0.531, p = 0.079).

| 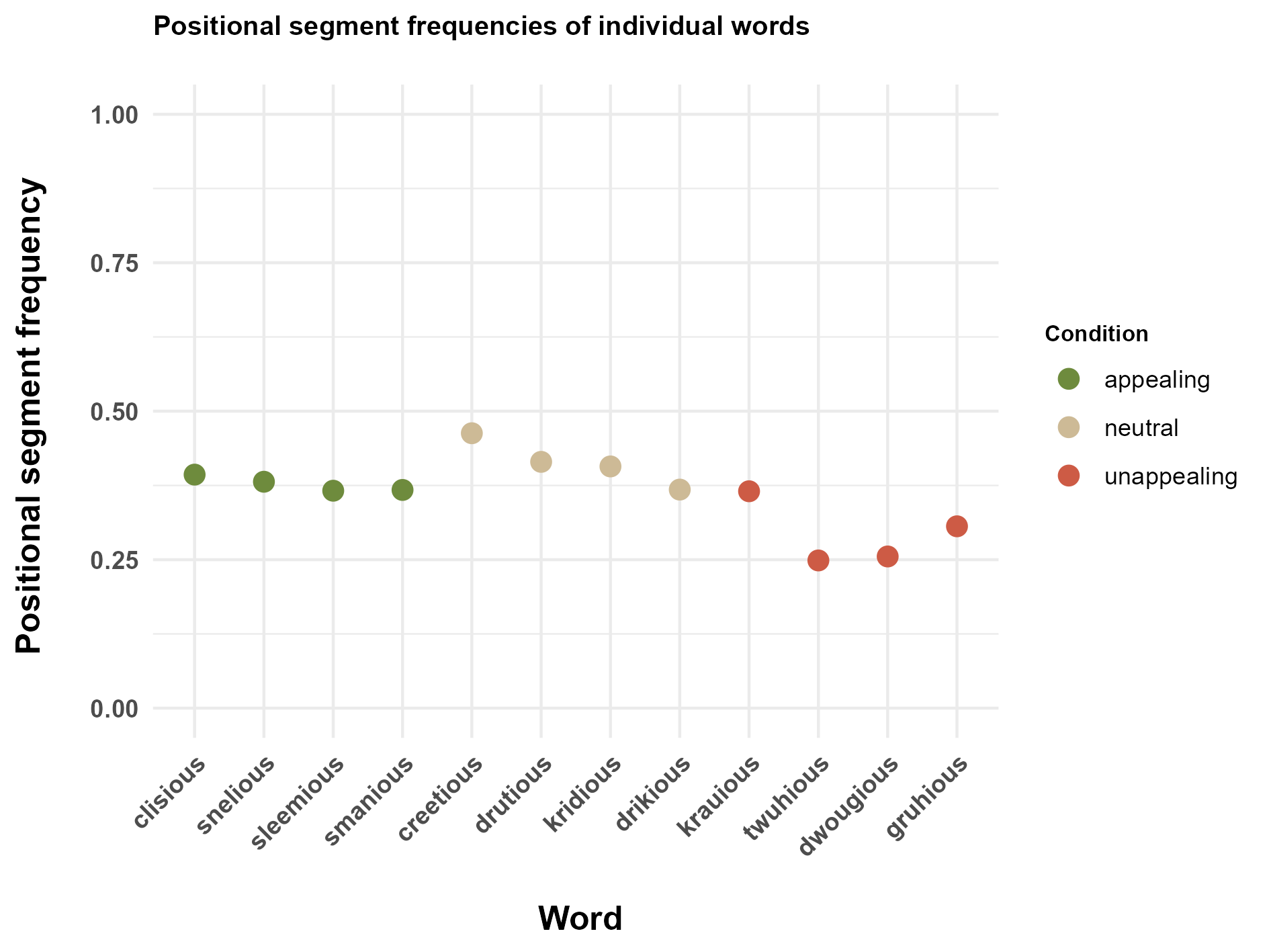 | 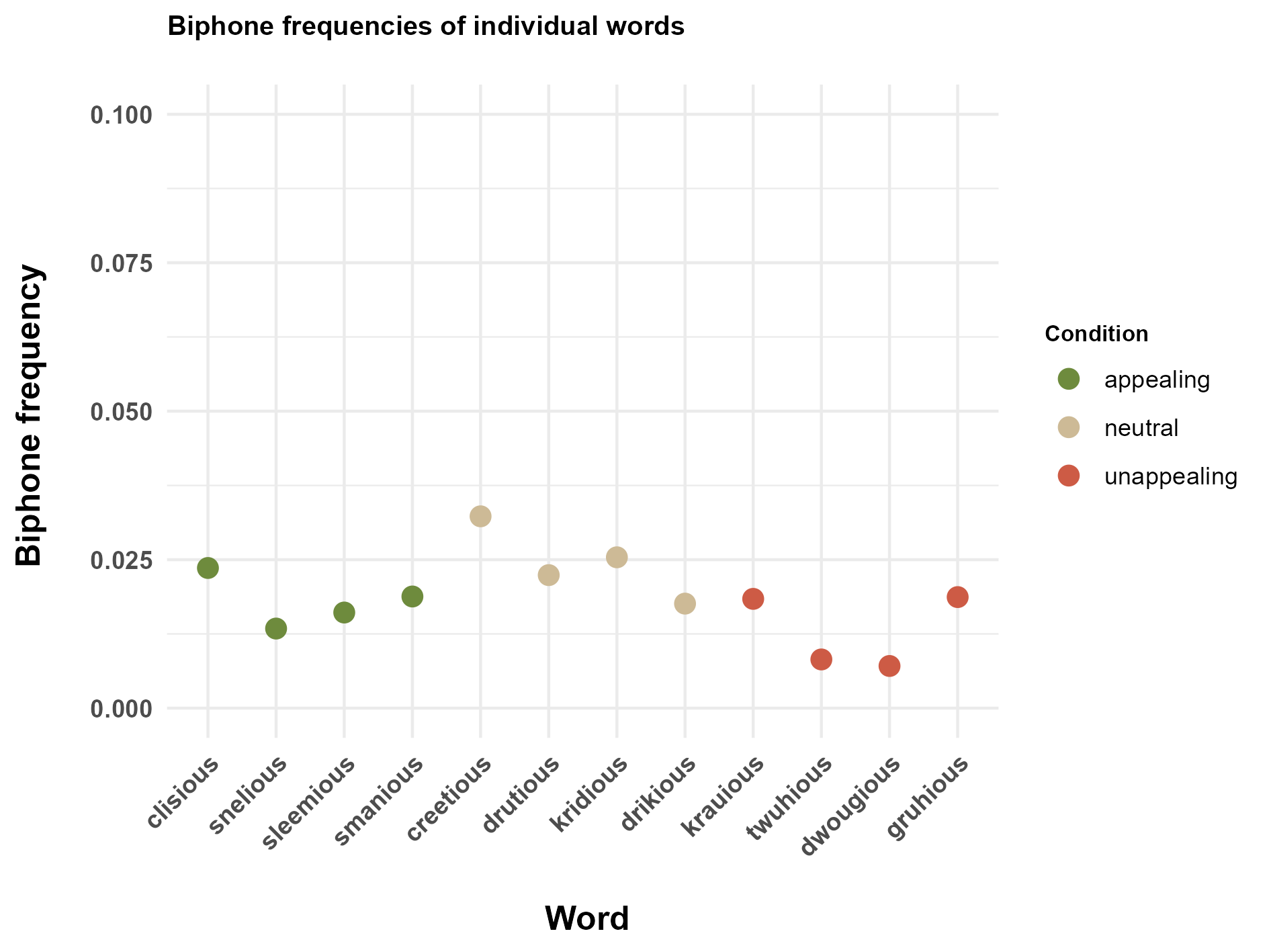 |
| --- | --- |

Figure A1. Positional segment frequencies (left) and biphone frequencies (right) of the pseudowords that were used as stimuli in the study.

To further support this, we included either positional segment frequency or biphone frequency as additional fixed effects in our CLMM (see Section 3.4.1 for details), with all other model parameters remaining unchanged. Both full models differed significantly from the respective null models (model including positional segment frequency: χ² = 39.10, df = 3, p < 0.001; model including biphone frequency: χ² = 39.36, df = 3, p < 0.001). Closer inspection of the model results revealed that neither positional segment frequency nor biphone frequency had a significant effect on ratings. The significant predictors remained the same as in the original model: both the appealing and the unappealing condition were rated significantly lower than the neutral condition (see Tab. A1 for the model including segment frequency and Tab. A2 for the model including biphone frequency). Thus, the observed effects are not driven by variations in positional segment or biphone frequency but instead reflect the influence of our experimental condition, i.e. aesthetic condition.

Table A1. Results of the Cumulative Link Mixed Model exploring the effects of aesthetic condition and positional segment frequency on the ratings of participants. Model formula: Rating ~ Condition + Trial number + Positional segment frequency + (Condition|Participant). The table reports estimated model coefficients (Estimate), standard errors (SE), z-values (z) and p-values (p) of the fixed effects, estimates, standard errors and z-values of the threshold coefficients, as well as variance, standard deviation (SD) and correlation coefficients (Corr) of the random effects.

| **Model coefficients** | **Estimate** | **SE** | **z** | **p** |  |
| --- | --- | --- | --- | --- | --- |
| Condition_appealing | -0.74 | 0.19 | -3.81 | <0.001 |  |
| Condition_unappealing | -0.74 | 0.18 | -4.12 | <0.001 |  |
| Trial number | -0.01 | 0.01 | -1.32 | 0.19 |  |
| Positional segment freq | 0.49 | 1.08 | 0.46 | 0.65 |  |
|  |  |  |  |  |  |
| **Threshold coefficients** | **Estimate** | **SE** | **z** |  |  |
| 1\|2 | -3.34 | 0.48 | -6.98 |  |  |
| 2\|3 | -1.78 | 0.48 | -3.74 |  |  |
| 3\|4 | -0.64 | 0.47 | -1.35 |  |  |
| 4\|5 | 0.50 | 0.47 | 1.06 |  |  |
| 5\|6 | 1.57 | 0.48 | 3.32 |  |  |
| 6\|7 | 2.97 | 0.48 | 6.20 |  |  |
|  |  |  |  |  |  |
| **Random effects** | **Term** | **Variance** | **SD** | **Corr** | **Corr** |
| Participant | Intercept | 1.77 | 1.33 |  |  |
|  | Condition_appealing | 2.71 | 1.65 | -0.26 |  |
|  | Condition_unappealing | 0.86 | 0.93 | -0.30 | 0.10 |

Table A2. Results of the Cumulative Link Mixed Model exploring the effects of aesthetic condition and biphone frequency on the ratings of participants. Model formula: Rating ~ Condition + Trial number + Biphone frequency + (Condition|Participant). The table reports estimated model coefficients (Estimate), standard errors (SE), z-values (z) and p-values (p) of the fixed effects, estimates, standard errors and z-values of the threshold coefficients, as well as variance, standard deviation (SD) and correlation coefficients (Corr) of the random effects.

| **Model coefficients** | **Estimate** | **SE** | **z** | **p** |  |
| --- | --- | --- | --- | --- | --- |
| Condition_appealing | -0.76 | 0.20 | -3.83 | <0.001 |  |
| Condition_unappealing | -0.79 | 0.15 | -5.15 | <0.001 |  |
| Trial number | -0.01 | 0.01 | -1.31 | 0.19 |  |
| Biphone freq | 2.18 | 7.56 | 0.29 | 0.77 |  |
|  |  |  |  |  |  |
| **Threshold coefficients** | **Estimate** | **SE** | **z** |  |  |
| 1\|2 | -3.52 | 0.26 | -13.53 |  |  |
| 2\|3 | -1.95 | 0.25 | -7.74 |  |  |
| 3\|4 | -0.81 | 0.25 | -3.23 |  |  |
| 4\|5 | 0.34 | 0.25 | 1.37 |  |  |
| 5\|6 | 1.42 | 0.25 | 5.67 |  |  |
| 6\|7 | 2.83 | 0.26 | 10.88 |  |  |
|  |  |  |  |  |  |
| **Random effects** | **Term** | **Variance** | **SD** | **Corr** | **Corr** |
| Participant | Intercept | 1.86 | 1.37 |  |  |
|  | Condition_appealing | 2.82 | 1.68 | -0.28 |  |
|  | Condition_unappealing | 0.87 | 0.93 | -0.32 | 0.12 |

### 1.2.2. Memory and recall

When examining the relationship between positional segment frequency or biphone frequency and word recall, Spearman rank correlation coefficients indicated no significant associations (positional segment frequency: rho = 0.225, p = 0.481; biphone frequency: rho = –0.095, p = 0.769).

To investigate this relationship further, we added either positional segment frequency or biphone frequency as fixed effects in our GLMM (see Section 3.4.2 for details). Due to convergence issues, the random slope of condition included in the original models was omitted, but all other model parameters remained unchanged. Consistent with the results from the rating data, neither positional segment frequency nor biphone frequency had a significant effect on recall. While both extended models differed significantly from their respective null models (model including positional segment frequency: χ² = 29.26, df = 3, p < 0.001; model including biphone frequency: χ² = 29.09, df = 3, p < 0.001), the additional predictors themselves were non-significant (see Tab. A3 and Tab. A4). Instead, the main effect of condition remained robust: words from both the neutral and the unappealing condition were recalled less often than those from the appealing condition.

To assess potential collinearity between predictors, we computed variance inflation factors (VIF) using the *car* package in R [[5]](https://www.zotero.org/google-docs/?luu85y). Both predictors were well below commonly used thresholds (positional segment frequency: condition: VIF = 1.33, segment frequency: VIF = 1.77; biphone frequency: condition: VIF = 1.17, biphone frequency: VIF = 1.37), indicating no problematic collinearity issues between predictors. Thus, condition effects on recall persisted even when controlling for positional segment and biphone frequency.

Table A3. Results of the generalized linear mixed model exploring the effects of aesthetic condition and positional segment frequency on word recall. Model formula: Words_recalled ~ Condition + Positional segment frequency + (1 | Participant). The table reports estimated model coefficients (Estimate), standard errors (SE), z-values and p-values (p).

| **Full Model** | **Estimate** | **SE** | **z** | **p** |
| --- | --- | --- | --- | --- |
| Intercept | -0.14 | 0.72 | -0.20 |  |
| Condition_neutral | -0.58 | 0.17 | -3.43 | <0.001 |
| Condition_unappealing | -0.75 | 0.22 | -3.41 | <0.001 |
| Positional segment freq | 0.79 | 1.87 | 0.42 | 0.67 |
|  |  |  |  |  |
| **Random effects** | **Term** | **Variance** | **SD** |  |
| Participant | Intercept | 0.69 | 0.83 |  |

Table A4. Results of the generalized linear mixed model exploring the effects of aesthetic condition and biphone frequency on word recall. Model formula: Words_recalled ~ Condition + Biphone frequency + (1 | Participant). The table reports estimated model coefficients (Estimate), standard errors (SE), z-values and p-values (p).

| **Full Model** | **Estimate** | **SE** | **z** | **p** |
| --- | --- | --- | --- | --- |
| Intercept | 0.14 | 0.28 | 0.51 |  |
| Condition_neutral | -0.55 | 0.18 | -3.14 | 0.002 |
| Condition_unappealing | -0.81 | 0.17 | -4.80 | <0.001 |
| Biphone freq | 0.88 | 13.34 | 0.07 | 0.95 |
|  |  |  |  |  |
| **Random effects** | **Term** | **Variance** | **SD** |  |
| Participant | Intercept | 0.69 | 0.83 |  |

# References

[1. Gathercole SE, Frankish CR, Pickering SJ, Peaker S. Phonotactic influences on short-term memory. *J Exp Psychol Learn Mem Cogn*. 1999;25: 84–95. doi:10.1037/0278-7393.25.1.84](https://www.zotero.org/google-docs/?tDW6ac)

[2. Roodenrys S, Hinton M. Sublexical or lexical effects on serial recall of nonwords? *J Exp Psychol Learn Mem Cogn*. 2002;28: 29–33. doi:10.1037/0278-7393.28.1.29](https://www.zotero.org/google-docs/?tDW6ac)

[3. Vitevitch MS, Luce PA, Charles-Luce J, Kemmerer D. Phonotactics and Syllable Stress: Implications for the Processing of Spoken Nonsense Words. *Lang Speech*. 1997;40: 47–62. doi:10.1177/002383099704000103](https://www.zotero.org/google-docs/?tDW6ac)

[4. Vitevitch MS, Luce PA. A Web-based interface to calculate phonotactic probability for words and nonwords in English. *Behav Res Methods Instrum Comput*. 2004;36: 481–487. doi:10.3758/BF03195594](https://www.zotero.org/google-docs/?tDW6ac)

[5. Fox J, Weisberg S. *An R companion to applied regression*. Third edition. Los Angeles London New Delhi Singapore Washington, DC Melbourne: SAGE; 2019.](https://www.zotero.org/google-docs/?tDW6ac)
