# Supplementary material for: Phonemic composition influences words’ aesthetic appeal and memorability
Source: PLoS One. 2025 Dec 3;20(12):e0336597. doi: 10.1371/journal.pone.0336597 (PMC12674511; doi:10.1371/journal.pone.0336597)
Supplement: Attachment — Submitted filename: RESPONSE_TO_REVIEWERS_2.pdf [file pone.0336597.s006.pdf]

Reviewer 2

Reviewer #2: Great job on very meticulous revisions! Thank you!

Abstract: Few lines are a bit confusing. “Nevertheless, pseudo-words designed to be highly appealing were recalled most frequently.” – maybe add “even though participants themselves did not rate them as highly appealing”. “Also, overall, recalled words received higher appeal ratings (add “from participants”) than non-recalled ones.”

We have made the changes accordingly.

Lit review: Minor cosmetic edits such as occasional lack of spacing, punctuations (double . .), pseudowords vs pseudo-words, some references in text are not in the alphabetic order, missing closing bracket.

We have made the changes accordingly.

p. 7: For “As a result, if speech sounds or phonotactic patterns differ in their aesthetic appeal, these differences in emotional arousal may have significant implications for our understanding of phonological short-term memory (Baddeley, 2003) and language acquisition. “ Maybe better: “As a result, if speech sounds or phonotactic patterns differ in their aesthetic appeal, these differences in emotional arousal may have significant implications for our understanding of phonological short-term memory FUNCTIONING (Baddeley, 2003) IN THE CONTEXT OF language acquisition. “

We have made the changes accordingly.

Results: In Table 2, what does the last column feature? I think the name of the column is missing.

The last column features the correlations between the two random slopes (condition_appealing, condition_unappealing). We have added “Corr” as a column heading.

Congratulations on an interesting paper!

Thanks a lot, and thanks for your thoughtful review!

Reviewer 3

Reviewer #3: This study aims to investigate the relationships between phonemes and aesthetic appeal on one hand, and between aesthetic appeal and memorization on the other hand, using pseudowords.

All the points I raised in the previous review round have been addressed. I am particularly happy to see that the effects of the experiment conditions (i.e., aesthetic appeal) remained robust and significant after controlling for phonotactic probability. I believe this additional analysis makes the main argument of this study much more convincing.

Thanks a lot!

I recommend this study for publication, with only a few minor stylistic comments:

1. Several occurrences of the word "phonemic" are missing a space afterward (e.g., "phonemicand", "phonemicquality", etc.) I would recommend adding a space after "phonemic" in each of these instances.

We have made the changes accordingly.

2. In the References section, some entries are indented while others are not. I just wanted to point this out, although such formatting issues may be checked and corrected at a later stage of publication.

We have made the changes accordingly.

Apart from these minor points, this study is well-structured, well-executed, clearly summarized, and persuasively argued. Thank you very much for this interesting work. I look forward to seeing further developments in this line of research.

Thanks a lot, and thanks for your thoughtful review!
