# Supplementary material for: Phonemic composition influences words’ aesthetic appeal and memorability
Source: PLoS One. 2025 Dec 3;20(12):e0336597. doi: 10.1371/journal.pone.0336597 (PMC12674511; doi:10.1371/journal.pone.0336597)
Supplement: Attachment — Submitted filename: PONE-D-25-04238_reviewer_VK.docx [file pone.0336597.s002.docx]

**PLOS ONE**

**Phonetic composition influences words’ aesthetic appeal and memorability**

--Manuscript Draft--

| **Manuscript Number:** | PONE-D-25-04238 |
| --- | --- |
| **Article Type:** | Research Article |
| **Full Title:** | Phonetic composition influences words’ aesthetic appeal and memorability |
| **Short Title:** | Phonetic composition influences words’ aesthetic appeal and memorability |
| **Corresponding Author:** | Theresa Matzinger Universitat Wien Vienna, AUSTRIA |
| **Keywords:** | phonaesthetics; aesthetic appeal; phonology; phonotactics; memory; artificial language learning |
| **Abstract:** | Positive emotional responses from pleasant experiences are known to enhance memory, yet the relationship between aesthetic appeal and linguistic memory remains understudied. To investigate this relationship, we designed pseudowords of varying appeal based on Crystal’s (1995) phoneme rankings. Native English-speaking participants actively memorized these pseudo-words and completed a free recall test, followed by two rounds of appeal ratings. Our results showed that, contrary to our predictions, pseudo-words designed to be of intermediate appeal were rated as more appealing than those designed to be highly appealing or unappealing. Nevertheless, pseudo-words designed to be highly appealing were recalled most frequently. Also, overall, recalled words received higher appeal ratings than non-recalled ones. These findings suggest that the phonetic and phonotactic composition of words may, indeed, have aesthetic value that correlates with words’ memorability. This encourages further exploration into how appeal interacts with other factors influencing linguistic cognition, including occurrence frequency or complexity. Our findings can inform applications in language learning, teaching, and marketing, while also offering theoretical contributions to our understanding of language evolution and change. |
| **Order of Authors:** | Theresa Matzinger |
|  | David Košić, BSc |
| **Additional Information:** |  |
| **Question** | **Response** |
| **Financial Disclosure**  Enter a financial disclosure statement that describes the sources of funding for the work included in this submission. Review the [submission guidelines](http://journals.plos.org/plosone/s/submission-guidelines#loc-financial-disclosure-statement) for detailed requirements. View published research articles from [*PLOS ONE*](https://journals.plos.org/plosone/) for specific examples.  This statement is required for submission and **will appear in the published article** if the submission is accepted. Please make sure it is accurate. | Yes |

| **Funded studies**  Enter a statement with the following details:   - Initials of the authors who received each award - Grant numbers awarded to each author - The full name of each funder - URL of each funder website - Did the sponsors or funders play any role in the study design, data collection and analysis, decision to publish, or preparation of the manuscript? |  |
| --- | --- |
| Did you receive funding for this work? |  |
| Please add funding details.  as follow-up to "**Financial Disclosure**  Enter a financial disclosure statement that describes the sources of funding for the work included in this submission. Review the [submission guidelines](http://journals.plos.org/plosone/s/submission-guidelines#loc-financial-disclosure-statement) for detailed requirements. View published research articles from [*PLOS ONE*](https://journals.plos.org/plosone/) for specific examples.  This statement is required for submission and **will appear in the published article** if the submission is accepted. Please make sure it is accurate. | This work was supported by a Disruptive Innovation Grant from the Austrian Academy of Sciences and the Austrian Science Fund (grant number: DI_2023- 108_MATZINGER_BEALP) awarded to Theresa Matzinger. |
| **Funded studies**  Enter a statement with the following details:   - Initials of the authors who received each award - Grant numbers awarded to each author - The full name of each funder - URL of each funder website - Did the sponsors or funders play any role in the study design, data collection and analysis, decision to publish, or preparation of the manuscript? |  |
| Did you receive funding for this work?" |  |
| Please select the country of your main research funder (please select carefully as in some cases this is used in fee calculation). | AUSTRIA - AT |

| as follow-up to "**Financial Disclosure**  Enter a financial disclosure statement that describes the sources of funding for the work included in this submission. Review the [submission guidelines](http://journals.plos.org/plosone/s/submission-guidelines#loc-financial-disclosure-statement) for detailed requirements. View published research articles from [*PLOS ONE*](https://journals.plos.org/plosone/) for specific examples.  This statement is required for submission and **will appear in the published article** if the submission is accepted. Please make sure it is accurate. |  |
| --- | --- |
| **Funded studies**  Enter a statement with the following details:   - Initials of the authors who received each award - Grant numbers awarded to each author - The full name of each funder - URL of each funder website - Did the sponsors or funders play any role in the study design, data collection and analysis, decision to publish, or preparation of the manuscript? |  |
| Did you receive funding for this work?" |  |
| **Competing Interests**  Use the instructions below to enter a competing interest statement for this submission. On behalf of all authors, disclose any [competing interests](http://journals.plos.org/plosone/s/competing-interests) that could be perceived to bias this work—acknowledging all financial support and any other relevant financial or non- financial competing interests.  This statement is required for submission and **will appear in the published article** if the submission is accepted. Please make sure it is accurate and that any funding sources listed in your Funding Information later in the submission form are also declared in your Financial Disclosure statement. | The authors have declared that no competing interests exist. |

| View published research articles from  [*PLOS ONE*](http://journals.plos.org/plosone/) for specific examples. |  |
| --- | --- |
| **NO authors have competing interests**  Enter: *The authors have declared that no competing interests exist.*  **Authors with competing interests**  Enter competing interest details beginning with this statement:  *I have read the journal's policy and the authors of this manuscript have the following competing interests: [insert competing interests here]* |  |
| * typeset |  |
| **Ethics Statement**  Enter an ethics statement for this submission. This statement is required if the study involved:   - Human participants - Human specimens or tissue - Vertebrate animals or cephalopods - Vertebrate embryos or tissues - Field research   Write "N/A" if the submission does not require an ethics statement.  General guidance is provided below. Consult the [submission guidelines](http://journals.plos.org/plosone/s/submission-guidelines#loc-guidelines-for-specific-study-types) for detailed instructions. **Make sure that all information entered here is included in the Methods section of the manuscript.** | The research protocol received approval from the Scientific Research Ethics Committee at the University of Vienna (reference number: 00998, 13.06.2023) and all participants provided written informed consent in adherence to the declaration of Helsinki. |

| **Format for specific study types**  **Human Subject Research (involving human participants and/or tissue)**   - Give the name of the institutional review board or ethics committee that approved the study - Include the approval number and/or a statement indicating approval of this research - Indicate the form of consent obtained (written/oral) or the reason that consent was not obtained (e.g. the data were analyzed anonymously)   **Animal Research (involving vertebrate animals, embryos or tissues)**   - Provide the name of the Institutional Animal Care and Use Committee (IACUC) or other relevant ethics board that reviewed the study protocol, and indicate whether they approved this research or granted a formal waiver of ethical approval - Include an approval number if one was obtained - If the study involved *non-human primates*, add *additional details* about animal welfare and steps taken to ameliorate suffering - If anesthesia, euthanasia, or any kind of animal sacrifice is part of the study, include briefly which substances and/or methods were applied   **Field Research**  Include the following details if this study involves the collection of plant, animal, or other materials from a natural setting:   - Field permit number - Name of the institution or relevant body that granted permission |  |
| --- | --- |
|  |  |
| **Data Availability**  Authors are required to make all data underlying the findings described fully available, without restriction, and from the time of publication. PLOS allows rare exceptions to address legal and ethical concerns. See the [PLOS Data Policy](http://journals.plos.org/plosone/s/data-availability) and [FAQ](http://journals.plos.org/plosone/s/data-availability#loc-faqs-for-data-policy) for detailed information. | Yes - all data are fully available without restriction |

| A Data Availability Statement describing where the data can be found is required at submission. Your answers to this question constitute the Data Availability Statement and **will be published in the article**, if accepted. |  |
| --- | --- |
| **Important:** Stating ‘data available on request from the author’ is not sufficient. If your data are only available upon request, select ‘No’ for the first question and explain your exceptional situation in the text box. |  |
| Do the authors confirm that all data underlying the findings described in their manuscript are fully available without restriction? |  |
| **Describe where the data may be found in full sentences. If you are copying our sample text, replace any instances of XXX with the appropriate details.**   - If the data are **held or will be held in a public repository**, include URLs, accession numbers or DOIs. If this information will only be available after acceptance, indicate this by ticking the box below. For example: *All XXX files are available from the XXX database (accession number(s) XXX, XXX.*). - If the data are all contained **within the manuscript and/or Supporting Information files**, enter the following: *All relevant data are within the manuscript and its Supporting Information files.* - If neither of these applies but you are able to provide **details of access elsewhere**, with or without limitations, please do so. For example:   *Data cannot be shared publicly because of [XXX]. Data are available from the XXX Institutional Data Access / Ethics Committee (contact via XXX) for researchers who meet the criteria for access to confidential data.*  *The data underlying the results presented in the study are available from (include the name of the third party* | We preregistered all hypotheses, study protocols, and analyses, including provisional R files, on the Open Science Framework (Preregistration date: 27 June, 2023; https://osf.io/npe2g/?view_only=f45c8373ecbd4c43abcc4ff4e00eb267).  All data and R files used for the analysis are available in the Open Science Framework repository and can be accessed at https://osf.io/npe2g/?view_only=f45c8373ecbd4c43abcc4ff4e00eb267. |

| *and contact information or URL).*   - This text is appropriate if the data are owned by a third party and authors do not have permission to share the data.   * typeset |  |
| --- | --- |
| Additional data availability information: | Tick here if the URLs/accession numbers/DOIs will be available only after acceptance of the manuscript for publication so that we can ensure their inclusion before publication. |

<https://www2.cloud.editorialmanager.com/pone/download.aspx?id=39114404&guid=5f27eef5-d8fa-4c74-aa26-6da72bcab653&scheme=1>Manuscript	[Click here to](https://www2.cloud.editorialmanager.com/pone/download.aspx?id=39114404&guid=5f27eef5-d8fa-4c74-aa26-6da72bcab653&scheme=1)

[access/download;Manuscript;PlosOne_Manuscript.docx](https://www2.cloud.editorialmanager.com/pone/download.aspx?id=39114404&guid=5f27eef5-d8fa-4c74-aa26-6da72bcab653&scheme=1)

# 1 Phonetic composition influences words’ aesthetic

2	appeal and memorability

3

#### 4 Theresa Matzinger^#^ ^1,2^ ^*^ & David Košić^#^ ^1^

5 # joint first authors
6 1 Department of English and American Studies, University of Vienna, Vienna, Austria
7 2 Vienna Cognitive Science Hub, University of Vienna, Vienna, Austria
8 * corresponding author: [theresa.matzinger@univie.ac.at](mailto:theresa.matzinger@univie.ac.at)
9 non-corresponding author: [david.kosic@univie.ac.at](mailto:david.kosic@univie.ac.at)
10 mailing address: Campus der Universität Wien, Hof 8.3, Spitalgasse 2-4, 1090 Wien, Austria 11

12

13	1. Abstract

14 Positive emotional responses from pleasant experiences are known to enhance memory, yet
15 the relationship between aesthetic appeal and linguistic memory remains understudied. To
16 investigate this relationship, we designed pseudowords of varying appeal based on Crystal’s
17 (1995) phoneme rankings. Native English-speaking participants actively memorized these
18 pseudo-words and completed a free recall test, followed by two rounds of appeal ratings. Our
19 results showed that, contrary to our predictions, pseudo-words designed to be of intermediate
20 appeal were rated as more appealing than those designed to be highly appealing or
21 unappealing. Nevertheless, pseudo-words designed to be highly appealing were recalled most
22 frequently. Also, overall, recalled words received higher appeal ratings than non-recalled ones.
23 These findings suggest that the phonetic and phonotactic composition of words may, indeed,
24 have aesthetic value that correlates with words’ memorability. This encourages further
25 exploration into how appeal interacts with other factors influencing linguistic cognition,
26 including occurrence frequency or complexity. Our findings can inform applications in
1 language learning, teaching, and marketing, while also offering theoretical contributions to our
2 understanding of language evolution and change. 3
4 **Keywords:** phonaesthetics, aesthetic appeal, phonology, phonotactics, memory, artificial
5 language learning

6

7

# 8 2. Introduction

9 Traditionally, aesthetics has been regarded as a philosophical discipline dedicated to studying
10 the artistic value of cultural artifacts [(Dewey, 2005; Vartanian & Nadal, 2022)](https://www.zotero.org/google-docs/?Dm4KrS). However, recent
11 studies suggest that aesthetic appeal is not limited to art but can also be observed in mundane
12 everyday experiences [(Isik & Vessel, 2021; Saito, 2007; Schindler et al., 2017; Vartanian &](https://www.zotero.org/google-docs/?JNtUgs)
13 [Nadal, 2022)](https://www.zotero.org/google-docs/?JNtUgs) such as the sounds of spoken language. This broader understanding of
14 aesthetics led to the emergence of the field of phonaesthetics, which is dedicated to studying
15 the aesthetic properties of speech sounds. Although the idea of phonaesthetics dates back to
16 the 1920s [(Jespersen, 2013)](https://www.zotero.org/google-docs/?Oqi8IW), it has since received little scholarly attention. Recently, research
17 has started to focus on the aesthetic appeal of languages as such [(Anikin et al., 2023; Kogan](https://www.zotero.org/google-docs/?ONicGl)
18 [& Reiterer, 2021; Winkler et al., 2023)](https://www.zotero.org/google-docs/?ONicGl), but systematic studies on the aesthetic appeal of
19 individual phonemes and phonotactic patterns remain scarce [(cf. Crystal, 1995)](https://www.zotero.org/google-docs/?VMvgyD).
20 This is surprising, since understanding if and how linguistic parameters differ in their aesthetic
21 appeal may have implications for several fields and disciplines. For example, it has
22 sociolinguistic relevance because it can help to explain why certain languages are perceived
23 as more beautiful than others [(Kogan & Reiterer, 2021; Winkler et al., 2023)](https://www.zotero.org/google-docs/?9SBUZH) and might in turn
24 help to understand and counteract language-based prejudices. Further, it might have
25 implications for language learning or language acquisition and may influence how frequently
26 certain words are used. This may in turn also affect, for example, language change [(Matzinger](https://www.zotero.org/google-docs/?8iJLfN)
27 [et al., 2021)](https://www.zotero.org/google-docs/?8iJLfN). To draw meaningful conclusions for those fields, it is essential to first establish
28 whether linguistic patterns vary in their aesthetic appeal at all, and if so, in what ways they
29 vary. Therefore, the main objective of our study is to determine which individual phonemes
30 and phonotactic patterns are perceived as more aesthetically appealing than others. A
31 secondary goal is to explore how these aesthetic preferences may interact with other cognitive
1 processes, such as memory, thereby expanding the scope of phonaesthetic research to
2 encompass more complex cognitive phenomena.
3 One of the few early studies that addressed the issue of aesthetic appeal of phonemes and
4 other linguistic features is of Crystal [(1995)](https://www.zotero.org/google-docs/?EcahqI). His analysis involved compiling lists of words
5 typically regarded as appealing, examining their phonemic makeup, and comparing the
6 frequency of phonemes in appealing words to their frequency in conversational English. He
7 found a tendency for appealing words to contain continuants (e.g., /l/, /s/), nasals (e.g., /m/,
8 /n/), front vowels (e.g., /i/, /e/), and diphthongs (e.g., /aɪ/, /eɪ/). Conversely, Crystal noted that
9 appealing words contained fewer “harsh” or abrupt sounds, such as plosives (e.g., /p/, /g/),
10 postalveolar fricatives (e.g., /ʃ/, /ʒ/), and affricates (e.g., /tʃ/, /dʒ/). While Crystal’s (1995) study
11 is an important first step in investigating the appeal of linguistic patterns, his study also comes
12 with many shortcomings. For example, Crystal’s [(1995)](https://www.zotero.org/google-docs/?gGzdz9) observations were based on
13 subjective lists of aesthetically pleasing words compiled from poets, lexicographers, and public
14 polls, which reflected individual preferences and biases rather than being derived from a
15 controlled, systematic analysis. Also, the lack of control for the semantic content of the words
16 in question may compromise potential conclusions about the inherent aesthetic qualities of
17 the linguistic features investigated, as the words’ meanings may have affected their aesthetic
18 perception. Thus, overall, to gain a more reliable understanding of the aesthetic appeal of
19 linguistic patterns, more comprehensive and systematic investigations are needed.
20 Though not explicitly focusing on *aesthetic* appeal, other studies that examined closely related
21 qualities such as valence and preference support the idea that different sounds and sound
22 patterns may also differ in their aesthetic appeal. For instance, Pogacar et al. [(2015)](https://www.zotero.org/google-docs/?XDxuHb) found
23 that top brand names often featured specific sounds such as front vowels, suggesting a link
24 between phonetic quality and consumer preference. Similarly, a study by Mooshammer et al.
25 ([2023)](https://www.zotero.org/google-docs/?eZBrz6) that examined conlangs found that fricatives were consistently associated with positive
26 meanings, while stops like /k/ and /g/ were linked to negative connotations. This is further
27 supported by Lev-Ari & McKay [(2023)](https://www.zotero.org/google-docs/?1ZhhTD) who found that pseudowords containing plosives were
28 more likely to be classified as swear words than words containing fewer or no plosives. Finally,
29 also, Körner & Rummer [(2023)](https://www.zotero.org/google-docs/?r1m7At) found that, across languages, words containing /i/ were
30 matched with positively connotated people and objects, while words containing /o/ or /u/ were
31 used for negatively connotated ones. This matches iconicity studies from consumer research
32 showing that brand names containing front vowels like /i/ were associated with positive traits
33 such as lightness and smallness, while names with back vowels like /o/ or /u/ conveyed traits
1 like heaviness, illustrating how phonetic qualities influence people’s perception of products
2 [(Klink & Athaide, 2012)](https://www.zotero.org/google-docs/?7GyyOs). Taken together, these studies suggest a link between phonetic quality
3 and aesthetic appeal, but it remains unclear if single phonemes are perceived as inherently
4 positive or negative, and how they contribute to the general appeal of words they constitute.
5 While there is already little systematic research on the aesthetic appeal of phonemes and
6 phonotactic patterns, there is even less research linking the appeal of speech parameters to
7 memory. One study indirectly addressing this topic is Matzinger et al. [(2021)](https://www.zotero.org/google-docs/?91ODvL), who found that
8 the aesthetic appeal of prosodic patterns correlates with their usefulness for segmenting
9 continuous speech into individual words. Although the study is not directly focusing on
10 memory, it suggests that aesthetically pleasing prosody may facilitate language processing
11 and learning.
12 Moreover, despite limited research on direct links between phonaesthetics and memory, there
13 is abundant evidence from other domains such as music and vision that suggest that aesthetic
14 appeal can positively impact human memory. Aesthetically appealing stimuli generally trigger
15 the human reward system [(Blood & Zatorre, 2001; Vartanian & Goel, 2004)](https://www.zotero.org/google-docs/?CznweE), prompting
16 humans to seek gratification from these stimuli and devote their attention to them rather than
17 to less rewarding ones [(Reppa & McDougall, 2015; Tamir & Robinson, 2007)](https://www.zotero.org/google-docs/?6fq3C4). This heightened
18 attention demands high cognitive involvement, ultimately enhancing the likelihood of memory
19 formation [(Payne & Kensinger, 2018)](https://www.zotero.org/google-docs/?Cod8vz). Furthermore, the cascade of positive emotions and
20 heightened arousal associated with appealing stimuli may contribute to memory consolidation,
21 ensuring these newly formed memories are stored long-term [(Blood & Zatorre, 2001;](https://www.zotero.org/google-docs/?y4oPeS)
22 [Vartanian & Goel, 2004)](https://www.zotero.org/google-docs/?y4oPeS). Even when humans do not actively seek or perceive rewarding
23 stimuli, they are still more likely to be memorized: arousing stimuli are better remembered
24 even if processed only peripherally [(Sharot et al., 2004)](https://www.zotero.org/google-docs/?tR0lhR). Conversely, less appealing or
25 "boring" stimuli significantly decrease memory performance and are likely to be quickly
26 forgotten [(Blondé et al., 2022)](https://www.zotero.org/google-docs/?IgX2EB). As a result, if speech sounds or phonotactic patterns differ in
27 their aesthetic appeal, these differences in emotional arousal may have significant implications
28 for our understanding of linguistic memory and language acquisition.
29 While the majority of evidence suggests that positive emotions boost memory, there are some
30 studies that claim otherwise. For example, research on negativity bias suggests that negative
31 experiences elicit more powerful emotions than neutral and positive experiences, leading to
32 more in-depth processing and faster encoding, consolidation, and retrieval of information
1 [(Baumeister et al., 2001; Rozin & Royzman, 2001)](https://www.zotero.org/google-docs/?RG2hD3). Seen from an evolutionary standpoint, this
2 tendency to retain negative experiences likely served an adaptive function since remembering
3 threats and dangers would be considered more critical than retaining positive memories
4 [(Kensinger & Corkin, 2003)](https://www.zotero.org/google-docs/?p8RVmQ). However, some scholars argue that both positive and negative
5 emotions enhance memory and cognitive processing through similar mechanisms,
6 emphasizing the role of emotional arousal as opposed to valence [(Anderson et al., 2006;](https://www.zotero.org/google-docs/?2UUsLG)
7 [Kousta et al., 2009)](https://www.zotero.org/google-docs/?2UUsLG). Thus, if phonaesthetic appeal affects word memory, it remains uncertain
8 whether pleasing or displeasing sounds will enhance recall more effectively.
9 To provide a more comprehensive picture of potential links between phonaesthetics and
10 memory, the aims of our study were twofold: (a) to test the aesthetic appeal of phonemes and
11 phonotactic patterns, thereby providing a more objective validation of Crystal’s ([1995](https://www.zotero.org/google-docs/?mW8Iot)) ratings,
12 and (b) to examine whether this appeal correlates with word memorability. For this reason, we
13 conducted an artificial language learning experiment, in which participants were exposed to
14 pseudowords that were designed to be appealing, neutral, or unappealing, as per Crystal
15 (1995). The experiment consisted of a rating task, in which participants had to judge how
16 appealing they found the pseudowords, and a free recall test, in which they had to recall the
17 pseudowords they had memorized. We used this data to validate and update Crystal’s (1995)
18 proposed rankings of phonetic appeal, and to correlate the word ratings with their recall
19 frequency.
20 We hypothesized that we would be able to confirm Crystal’s (1995) ratings and predicted that
21 pseudowords containing sounds that ranked highly in Crystal’s hierarchy (from now on called
22 “appealing words” or the “appealing condition”) would receive higher ratings of aesthetic
23 appeal than words containing sounds that ranked lowly in Crystal’s hierarchy (from now on
24 called “unappealing words” or the “unappealing condition”), with pseudowords containing
25 sounds from the middle of Crystal’s hierarchy (from now on called “neutral words” or the
26 “neutral condition”) receiving intermediate ratings.
27 Regarding memory, we hypothesized that appeal would influence word memorization.
28 Specifically, we predicted that, if high appeal positively influences memory, words from the
29 appealing condition should be recalled most frequently and, conversely, words from the
30 unappealing condition should be recalled least frequently. Words from the neutral condition
31 should fall somewhere in between. Alternatively, if unappealing words are more memorable,
32 we would expect the opposite effect. If the extremes of the emotional scale, irrespective of
1 their valence, are crucial for memory formation, appealing and unappealing pseudowords
2 should be recalled equally often but more often than neutral stimuli.

3

# 4 3. Methods

## 5 3.1. Experimental conditions, procedure and setting

6 To test whether the phonetic makeup of words influenced their perceived aesthetic appeal,
7 and whether this influenced the words’ memorability, we conducted an experiment that was
8 divided into three phases: stimulus presentation, stimulus recall, and stimulus rating (Fig. 1).
9 In the first phase, the stimulus presentation phase, participants were exposed to a set of stimuli
10 that were designed to be either appealing, neutral or unappealing (based on Crystal, 1995;
11 see Section 3.3. for more details). Participants were exposed to four stimuli in each of the
12 three conditions visually and audibly. The stimuli were displayed on the screen in random
13 order with each stimulus appearing six times for three seconds to help aid memorization. Audio
14 recordings of the stimuli’s pronunciation played automatically in the background, with the audio
15 starting immediately as a written stimulus appeared on the screen. Participants were
16 instructed to orally repeat each stimulus to ensure full concentration on the task and further
17 promote memorability. After all stimuli had been presented, the setup automatically proceeded
18 to the second phase, the stimulus recall phase. 19

20

21 **Figure 1. Experimental setup**. The experiment consisted of three phases: (1) *Stimulus*
22 *Presentation*, where participants memorized visually and audibly presented stimuli; (2)
1 *Stimulus Recall*, where they typed the stimuli that they recalled; and (3) *Stimulus Rating*,
2 where they rated each stimulus on a 7-point Likert scale for appeal. 3
4 In the stimulus recall phase, participants were prompted to recall and type the words that they
5 had memorized. They were informed that they should enter all words that they had
6 remembered, even if they were not fully certain about their answers. 7
8 In the third phase, the stimulus rating phase, participants were instructed to rate the appeal of
9 each stimulus on a 7-point Likert scale ranging from “unappealing” to “appealing”. There were
10 two separate rating rounds, i.e. each stimulus was presented twice to check for consistency
11 of the ratings within individuals. Within the two rating rounds, the stimuli were displayed in
12 random order. Participants gave their ratings by pressing a corresponding number key on the
13 keyboard. To elicit the participants’ ratings, we used the prompt “Rate the following word’s
14 appeal”. We decided to not further instruct or brief the participants on the term “appeal”
15 because, even after providing definitions, it would remain a very subjective term that is prone
16 to individual interpretations [(Aleem et al., 2019; Vashi & Quay, 2015)](https://www.zotero.org/google-docs/?G9wn1z), and we intended to elicit
17 intuitive spontaneous responses rather than responses biased or superimposed by potential
18 instructions. After completing the ratings, the participants were debriefed, thanked for their
19 participation and the experiment was concluded.
20 The experiment was conducted online, and participants were instructed to participate from a
21 quiet room without distractions. Participants were also required to use headphones to ensure
22 proper presentation of auditory stimuli. 23

## 24 3.2. Participants

25 The study comprised a sample of 100 native English speakers (47 male, 48 female, 5 other;
26 mean age 29.35 ± SD 10.21 years). We did not make any restrictions regarding the variety of
27 English spoken. The choice of our sample size was based on the results of a power analysis
28 (using the *pwr* package in R; Champely 2020), with a desired power of 0.8, a significance level
29 of 0.05 and an assumed small effect size (Cohen 1988). Participants were recruited through
30 the crowdsourcing platform Prolific ([www.prolific.com](http://www.prolific.com/), 2024) and received a remuneration of
31 £ 1.0 for their approximate 10-minute participation. The research protocol received approval
32 from the Scientific Research Ethics Committee at the University of Vienna (reference number:
1 00998; 13.06.2023) and all participants provided written informed consent in adherence to the
2 declaration of Helsinki. The main data collection happened after receiving ethical approval
3 (start: 27.06.2023, end: 03.01.2024).

4

## 5 3.3. Stimulus Design

6 We designed twelve English-sounding pseudo-words to use as stimuli in our study. We opted
7 for pseudo-words that did not resemble real English words to prevent the words’ appeal from
8 being influenced by semantic content or connotations. Likewise, the English sounds of the
9 words should prevent negative effects on appeal due to unfamiliarity. All pseudo-words were
10 trisyllabic and followed an identical structure: they started with a consonant cluster, followed
11 by a vowel and single consonant, and ended in the suffix “-ious” (CCVC-ious). We ensured
12 that the structure of all pseudo-words followed English phonotactics.
13 As outlined above, all stimuli were designed to be either “appealing”, “neutral” or
14 “unappealing”, by using consonants and vowels that ranked differently in Crystal’s (1995)
15 phonaesthetic hierarchy to fill the slots in the above-describe structure. In Crystal’s analysis,
16 so-called “N values” were used to measure the number of occurrences of specific phonemes
17 in the set of words that were regarded as appealing by his study participants. We utilized
18 phonemes with higher N values for stimuli of the appealing condition, and, conversely,
19 phonemes with lower N values for stimuli of the unappealing condition. For the neutral
20 condition, we used phonemes with intermediate N-values. We then calculated the average N
21 value of all phonemes in each stimulus (Tab. 1). For our conditions to be distinct in their
22 appeal, we ensured that the average ratings of the stimuli in each condition differed sufficiently
23 from the other conditions. To present the stimuli visually, each stimulus was assigned a
24 matching written form.
25 Here, we illustrate the stimulus design process, using the example of the pseudo-word
26 ”clisious.“ The consonants /k/ and /l/ were picked from the top of Crystal’s (1995) list to form
27 the first cluster of the stimulus. The vowel /ɪ/, also on top of Crystal’s (1995) hierarchy, was
28 selected to complete the first syllable. Another highly rated consonant, /s/, and the suffix “-
29 ious” were added to complete the stimulus. Finally, the written form “clisious” was assigned to
30 represent the spoken, phonetic form of the stimulus created earlier. The list of all stimuli and
31 their structure is outlined in Table 1. A microphone (Tonor Q9) was used to record the pseudo-
1 words, with the initial syllable being stressed in each stimulus. To keep variation in voice
2 quality, speech tempo and intonation to a minimum, all stimuli were recorded by the same
3 male speaker in a single recording session. All recordings were checked for clipping and
4 inaccuracies in Audacity (Audacity Team, 2022) and amplified and normalized to constant
5 volume.

6

| **Condition** | **Phonetic Structure** | | | | | **Written Form** |
| --- | --- | --- | --- | --- | --- | --- |
|  | **N Value** | | | | | **Average N Value** |
| **APPEALING** | **k** | **l** | **ɪ** | **s** | **ɪous** | **clisious** |
|  | 28 | 59 | 49 | 35 | - | **42.75** |
|  | **s** | **n** | **e** | **l** | **ɪous** | **snelious** |
|  | 35 | 33 | 24 | 59 | - | **37.75** |
|  | **s** | **l** | **iː** | **m** | **ɪous** | **sleemious** |
|  | 35 | 59 | 14 | 40 | - | **37.00** |
|  | **s** | **m** | **æ** | **n** | **ɪous** | **smanious** |
|  | 35 | 40 | 24 | 33 | - | **33.00** |
| **NEUTRAL** | **k** | **r** | **iː** | **t** | **ɪous** | **creetious** |
|  | 28 | 29 | 14 | 26 | - | **24.25** |
|  | **k** | **r** | **aɪ** | **d** | **ɪous** | **kridious** |
|  | 28 | 29 | 13 | 24 | - | **23.5** |
|  | **d** | **r** | **aɪ** | **k** | **ɪous** | **drikious** |
|  | 24 | 29 | 13 | 28 | - | **23.5** |
|  | **d** | **r** | **ʌ** | **t** | **ɪous** | **drutious** |
|  | 24 | 29 | 12 | 26 | - | **22.75** |
| **UNAPPEALING** | **k** | **r** | **aʊ** | **w** | **ɪous** | **krauious** |
|  | 28 | 29 | 1 | 8 | - | **16.5** |
|  | **t** | **w** | **uː** | **h** | **ɪous** | **twuhious** |
|  | 26 | 8 | 7 | 6 | - | **11.75** |
|  | **d** | **w** | **aʊ** | **g** | **ɪous** | **dwougious** |
|  | 24 | 8 | 7 | 7 | - | **11.5** |
|  | **g** | **r** | **uː** | **h** | **ɪous** | **gruhious** |

|  | 7 | 29 | 1 | 6 | - | **10.75** |
| --- | --- | --- | --- | --- | --- | --- |

2

1

3 **Table 1. List of stimuli used in the experiment.** The first column shows the experimental
4 condition. The second column shows the phonetic make-up of the pseudo-words based on
5 the N values (corresponding to appeal) assigned to each phoneme by Crystal (1995). The
6 third column shows the written form of the pseudo-words and their average N values, based
7 on the N values of the individual phonemes.

8

## 9 3.4. Analyses

### 10 3.4.1. Aesthetic appeal

11 To test whether the aesthetic perception of the pseudo-words was influenced by the
12 phonemes that they contained, i.e. whether words in our three aesthetic conditions would
13 receive different ratings, we employed a Cumulative Link Mixed Model (CLMM;
14 Christensen, 2018, 2019; Christensen & Brockhoff, 2013), using Laplace approximation [(Joe,](https://www.zotero.org/google-docs/?pW92eP)
15 [2008; Pinheiro & Bates, 1995)](https://www.zotero.org/google-docs/?pW92eP). To account for the ordinal nature of the Likert scale ratings and
16 as CLMMs are specifically designed for analyzing ordered categorical data (Christensen,
17 2019), we employed a CLMM instead of a linear mixed-effects model. In this model, *aesthetic*
18 *condition,* which was the predictor of our main interest, was included as a fixed effect.
19 Additionally, we included *trial number* as a fixed effect. It served as a control predictor to
20 ensure that ratings were not influenced by the point in the experiment when a particular
21 stimulus occurred. We also entered a random intercept of *participant* into the model. To
22 prevent inflated type I error rates, each model included a random slope [(Barr, 2013)](https://www.zotero.org/google-docs/?Nst7HK) of
23 *aesthetic condition* within *participant*. An initial model that also included a random slope of
24 *word* within *participant* did not converge. The sample size for the model was 2,400 data points
25 (100 participants each provided 2 ratings for stimuli across 3 aesthetic conditions that were
1 comprised of 4 pseudo-words each). The neutral aesthetic condition was set as the reference
2 level in the model.
3 The model was fitted in R (version 4.3.0; R Development Core Team, 2023), using the function
4 *clmm* of the R-package “ordinal” (version 2022.11.16; Christensen, 2023).
5 We employed a likelihood ratio test to test the overall significance of the full model, comparing
6 it to a null model comprising only the random effects and the control predictor of *trial number*
7 (R function *anova*; Dobson, 2002).

8

### 9 3.4.2. Memory and recall

10 Memory was assessed by counting how many pseudo-words participants correctly recalled
11 during the recall phase of the experiment. As participants occasionally made typos when
12 recalling the pseudo-words (e.g., typing "cliisious" instead of "clisious" or "grhuious" instead of
13 "gruhious"), we implemented a flexible criterion to account for minor deviations. To achieve
14 this, we applied the Jaro-Winkler distance [(Cohen et al., 2003; Jaro, 1989; W. E. Winkler,](https://www.zotero.org/google-docs/?JoIoh5)
15 [1990)](https://www.zotero.org/google-docs/?JoIoh5) with a threshold of 0.15, which allowed us to match participants' written responses to
16 the closest correct response. This threshold was specific enough to correct for small,
17 typographical errors (roughly 1-2 letters off) but strict enough to avoid counting responses that
18 bore little resemblance to the original stimulus. By doing so, we ensured that responses that
19 were intuitive matches to the original stimuli were counted, while responses with significant
20 differences were excluded.
21 To test if the participants’ word recall was influenced by the phonemes that the words
22 contained, i.e. by the aesthetic condition that the words belonged to, we fitted a Generalized
23 Linear Mixed Effects Model [(Baayen, 2008)](https://www.zotero.org/google-docs/?VWDPl9) with a logit link function [(McCullagh & Nelder,](https://www.zotero.org/google-docs/?dkBRrM)
1 [1989)](https://www.zotero.org/google-docs/?dkBRrM). The model included a fixed effect of *aesthetic condition*, a random intercept effect of
2 *participant* and a random slope of *aesthetic condition* within *participant* [(Barr, 2013)](https://www.zotero.org/google-docs/?4RzzG8). We set
3 the appealing aesthetic condition as the reference level in the model. To test if the participants’
4 recall of words was correlated with their own ratings of those words, we fitted another
5 Generalized Linear Mixed Effects Model with a logit link function that included a fixed effect of
6 the word’s *rating* (each participant’s mean rating of each word from rounds 1 and 2), a random
7 intercept effect of *participant* and a random slope of *rating* within *participant*. The dependent
8 variable in both models was whether the participants had correctly recalled the respective
9 word (with the levels “yes” and “no”). The sample size of both models was 1200 data points
10 (100 individuals could recall 4 words in 3 aesthetic conditions each), 523 of which were
11 correctly recalled words.
12 The models were calculated in R (version 4.3.0; R Development Core Team, 2023), using the
13 function *glmer* of the R-package *lme4* (version 1.1-33; [Bates et al., 2015](https://www.zotero.org/google-docs/?ZEcCDB)). To test the overall
14 significance of *aesthetic condition* or *rating*, respectively, on word recall, we employed
15 likelihood ratio tests to compare our full models to null models that did not include *condition* or
16 *rating*, respectively, but only the random intercept of *participant* and the random slope of
17 *condition* or *rating*, respectively, within *participant* (R function *anova*; Dobson, 2002). To
18 evaluate the goodness-of-fit of our models, we computed both marginal and conditional R2
19 values for our full models [(Nakagawa & Schielzeth, 2013)](https://www.zotero.org/google-docs/?lB3peZ). The marginal R2 (R2m) reflects the
20 variance accounted for solely by the fixed effects, while the conditional R2(R2c) represents the
21 variance explained by both the fixed and the random effects combined. Thus, these measures
22 give an indication of the model’s overall effect size. We calculated both R2 values using the
23 *r.squaredGLMM* function from the *MuMIn* package, using the “theoretical” method (Bartón,
24 2024). We preregistered all hypotheses, study protocols, and analyses, including provisional
1 R files, on the Open Science Framework (Preregistration date: 27 June, 2023;
2 <https://osf.io/npe2g/?view_only=f45c8373ecbd4c43abcc4ff4e00eb267>).

3

4

# 5 4. Results

6

## 7 4.1. Aesthetic appeal

8 Overall, the full model (Tab. 2) differed significantly from the null model, which shows that the
9 aesthetic condition, i.e. the phonemes and phonotactic patterns that a word consisted of,
10 influenced as how aesthetically pleasing participants rated the words (likelihood ratio test: χ2

11	= 38.59, df = 2, p < 0.001; Fig. 2A). However, in contrast to our predictions, words in the neutral

12 condition received higher ratings (mean rating: 4.17 ± 0.11) than words in the appealing (mean
13 rating: 3.60 ± 0.13) and unappealing (mean rating: 3.56 ± 0.12) conditions (see model results
14 in Tab. 2 and non-overlapping 95% confidence intervals in Fig. 2B). There was no significant
15 difference in the ratings of the words in the appealing and unappealing condition (see model
16 results in Tab. 2 and overlapping 95% confidence intervals in Fig. 2B). Thus, our results did
17 not replicate Crystal’s (1995) rankings of the aesthetic appeal of phonemes that would have
18 predicted the aesthetically appealing condition to get the highest ratings. This pattern is also
19 reflected by the ratings for individual words, which resemble an inverted U-shaped curve (Fig.
20 2C), with the most and least appealing words according to Crystal’s (1995) ranking receiving
21 the lowest ratings, and neutral words receiving the highest ratings. The only exception to this
22 curve is the word *clisious*, which was designed to be the most appealing, and received higher
23 ratings than expected based on the inverted U-shaped pattern. There was no significant effect
1 of *trial number* on the ratings (Tab. 2), indicating that experiment progress did not lead to a
2 consistent change of the participants’ ratings.

3

#### 4 Table 2. Results of the Cumulative Link Mixed Model exploring the effects of aesthetic

5 **condition on the ratings of participants.** The table reports estimated model coefficients
6 (Estimate), standard errors (SE), z-values (z) and p-values (p) of the fixed effects, estimates,
7 standard errors and z-values of the threshold coefficients, as well as variance, standard
8 deviation (SD) and correlation coefficients (Corr) of the random effects.

9

| **Model coefficients** | **Estimate** | **SE** | **z** | **p** |  |
| --- | --- | --- | --- | --- | --- |
| Condition_appealing | -0.76 | 0.19 | -4.05 | <0.001 |  |
| Condition_unappealing | -0.80 | 0.13 | -6.15 | <0.001 |  |
| Trial number | -0.01 | 0.01 | -1.56 | 0.12 |  |
|  |  |  |  |  |  |
| **Threshold coefficients** | **Estimate** | **SE** | **z** |  |  |

| 1\|2 | -3.53 | 0.18 | -19.28 |  |  |
| --- | --- | --- | --- | --- | --- |
| 2\|3 | -1.98 | 0.17 | -11.65 |  |  |
| 3\|4 | -0.85 | 0.17 | -5.13 |  |  |
| 4\|5 | 0.28 | 0.17 | 1.72 |  |  |
| 5\|6 | 1.36 | 0.17 | 8.05 |  |  |
| 6\|7 | 2.74 | 0.18 | 15.08 |  |  |
|  |  |  |  |  |  |
| **Random effects** | **Term** | **Variance** | **SD** | **Corr** |  |
| Participant | Intercept | 1.77 | 1.33 |  |  |
|  | Condition_appealing | 2.68 | 1.64 | -0.28 |  |
|  | Condition_unappealing | 0.88 | 0.94 | -0.31 | 0.12 |

1

2

**C**

1

#### 2 Figure 2. Beauty ratings of words that included either appealing, neutral or unappealing

3 **phonemes (according to Crystal, 1995).** A. Boxes depict medians and quartiles, and
4 whiskers depict minimum and maximum values. Violin shapes around the boxes show the
5 distributions of the ratings. The width of the violin shapes at a given y coordinate corresponds
6 to the number of ratings in this region. B. Dots denote mean values and error bars 95%
7 confidence intervals. Non-overlapping confidence intervals indicate significant differences
8 between the conditions [(Cumming & Finch, 2005)](https://www.zotero.org/google-docs/?jXT9qf). C. Mean values and 95% confidence
1 intervals of the ratings of the individual pseudo-words. Mean ratings resemble an inverted U-
2 shaped curve.

3

4 A Spearman rank correlation analysis was performed to examine the association between the
5 ratings obtained in round 1 and round 2 of the rating task. This revealed a statistically
6 significant positive correlation (rho = 0.66, p < 0.001), indicating that participants who gave
7 high ratings for particular words in rating round 1 were likely to also give high ratings for those
8 words in rating round 2 (Fig. 3).

9

10

#### 1 Figure 3. Scatterplot illustrating the correlation between ratings in rating round 1 and

2 **rating round 2 of the experiment.** Each point represents an individual participant, with the
3 x-axis corresponding to ratings in round 1 and the y-axis to ratings in round 2. Gray areas
4 around the regression line denote 95% confidence regions.

5

## 6 4.2. Memory and recall

7 The comparison of the full and the null model indicated an effect of aesthetic condition on word
8 recall (likelihood ratio test: χ2 = 29.91, df = 2, p < 0.001; effect size for the full model: R2m =
9 0.03; R2c = 0.22). More precisely, the full model (Tab. 3) showed that in the appealing condition
10 (average percentage of recalled words per participant: 53.25 ± CI 5.63 %; Fig. 4A, Fig. 4B),
11 participants recalled more words than in the neutral (average percentage of recalled words
12 per participant: 41.5 ± CI 5.29 %) and unappealing conditions (mean percentage of recalled
13 words per participant: 36.00 ± CI 6.04 %). This is also reflected in the percentage of
14 participants remembering the individual words of each condition (Fig. 4C, with the pseudo-
15 words “smanious” and “creetious” showing slight deviations from the general pattern within
16 their respective conditions).

17

#### 18 Table 3. Results of the generalized linear mixed effects model exploring the effects of

19 **aesthetic condition on word recall.** The table reports estimated model coefficients
20 (Estimate), standard errors (SE), z-values and p-values (p).

| **Full Model** | **Estimate** | **SE** | **z** | **p** |
| --- | --- | --- | --- | --- |

| **Intercept** | 0.15 | 0.13 | 1.15 |  |
| --- | --- | --- | --- | --- |
| **Condition_neutral** | -0.53 | 0.15 | -3.47 | <0.001 |
| **Condition_unappealing** | -0.87 | 0.17 | -5.26 | <0.001 |

1

**A**

**B**

**C**

1

#### 2 Figure 4. Per-participant-recall of words that included either appealing, neutral or

3 **unappealing phonemes (according to Crystal, 1995; in percent).** A. Boxes depict medians
4 and quartiles, and whiskers depict minimum and maximum values. Violin shapes around the
5 boxes show the distributions of the percentages of recalled words per participant. The width
6 of the violin shapes at a given y coordinate corresponds to the number of ratings in this region.
7 B. Dots denote mean values and error bars 95% confidence intervals. Non-overlapping
8 confidence intervals indicate significant differences between the conditions [(Cumming &](https://www.zotero.org/google-docs/?51O0Fn)
9 [Finch, 2005)](https://www.zotero.org/google-docs/?51O0Fn). C. Percentage of participants recalling the individual pseudo-words.

10

1

2 Our second generalized linear mixed effects model, which tested the effect of the participants’
3 actual ratings on word recall, revealed a (non-significant) tendency for the ratings to correlate
4 with word recall (likelihood ratio test: χ2 = 3.51, df = 1, p = 0.061; effect size for the full model:
5 R2m = 0.01; R2c = 0.18): participants tended to be more likely to recall those words that they
6 also rated more highly (Tab. 4). Also, on average, recalled words received significantly higher
7 ratings (3.93 ± CI 0.11) than non-recalled words (3.66 ± 0.09; Fig. 5, see non-overlapping
8 confidence intervals).

9

#### 10 Table 4. Results of the generalized linear mixed model exploring the effects of rating on

11 **word recall.** The table reports estimated model coefficients (Estimate), standard errors (SE),
12 z-values and p-values (p).

| **Full Model** | **Estimate** | **SE** | **z** | **p** |
| --- | --- | --- | --- | --- |
| **Intercept** | -0.65 | 0.21 | -3.12 |  |
| **Rating** | 0.09 | 0.05 | 1.90 | 0.058 |

13

14

1

2 **Figure 5. Mean ratings of recalled and non-recalled words.** Dots denote mean values and
3 error bars 95% confidence intervals. Non-overlapping confidence intervals indicate significant
4 differences between the conditions [(Cumming & Finch, 2005)](https://www.zotero.org/google-docs/?oZLA4A).

# 5 5. Discussion

6 This study demonstrates that words with different phonetic compositions are perceived
7 differently in terms of aesthetic appeal and that this perceived appeal correlates with word
8 memory. However, not all of our results aligned with our specific predictions.

## 1 5.1. Ratings of aesthetic appeal

2 Contrary to our expectations, words that we had designed to be appealing based on the criteria
3 proposed by Crystal (1995) were not rated as the most appealing by our participants. Instead,
4 they received similar ratings as words designed to be unappealing. Interestingly, words
5 designed to have intermediate appeal received the highest ratings. A closer examination of
6 the individual stimuli revealed, overall, an inverted U-shaped pattern in the ratings. As a result,
7 we were unable to replicate Crystal’s criteria for designing appealing words.
8 Several factors may explain these findings. First, Crystal’s (1995) rankings of phonemes,
9 which we used to design our stimuli and experimental conditions, were based on anecdotal
10 reports and observations. Moreover, Crystal (1995) focused on existing English words with
11 semantic meaning, which likely influenced the perception of their appeal. In contrast, our study
12 employed an artificial language, eliminating semantic associations and encouraging
13 participants to focus more directly on the intrinsic appeal of speech sounds and phonotactic
14 patterns. This methodological difference may have contributed to the divergence in results.
15 Secondly, the phonetic composition of the words in Crystal’s (1995) study might have
16 interacted with other factors, such as syllable count or word stress, which may have influenced
17 the perceptions of appeal but were not described in detail in Crystal’s work. We opted for a
18 controlled design with constant syllable counts and word stress. It is possible that interactions
19 between individual speech sounds, their combinations, and other factors influenced the
20 ratings, leading to the fact that Crystal’s (1995) ranking diverged from our findings.
21 Additionally, our results align with the inverted U-curves described in research linking appeal
22 with familiarity, which suggests that familiarity can strongly influence perceptions of appeal
23 [(Berlyne, 1970; Sluckin et al., 1980)](https://www.zotero.org/google-docs/?nssJBy). Because our stimulus design was based on Crystal’s
1 (1995) rankings of appeal, we were unable to fully control for the occurrence frequency of
2 phonemes in the participants’ L1, English. Consequently, many of the phonemes that received
3 high ratings in Crystal’s (1995) study and were therefore placed in our “appealing” condition
4 (e.g., /n/, /s/, /l/), are also highly frequent in the English language [(Crystal, 1995;](https://www.zotero.org/google-docs/?rnKgvH) [Hayden,](https://www.zotero.org/google-docs/?rnKgvH)
5 [1950; Mines et al., 1978)](https://www.zotero.org/google-docs/?rnKgvH). The inverted U-curve theory posits that items with very high
6 frequency can reduce appeal due to overstimulation and boredom, while very rare or
7 unfamiliar items may evoke uncertainty and discomfort. As a result, both highly frequent and
8 highly infrequent sounds or patterns may receive lower ratings. In contrast, neutral stimuli—
9 those that strike a balance between familiarity and novelty—may achieve higher appeal,
10 potentially explaining the high ratings of words in our neutral condition. Still, familiarity alone
11 cannot fully account for our results, as Crystal’s (1995) rankings of appeal and occurrence
12 frequency do not align perfectly. For instance, /ð/, which is relatively frequent in English (falling
13 in the upper-mid range of the frequency hierarchy), is ranked as having very low aesthetic
14 appeal in Crystal's (1995) observations.
15 Finally, on a more speculative note, cultural aesthetic preferences may also fluctuate over time
16 [(Jacobsen & Beudt, 2017)](https://www.zotero.org/google-docs/?RfTfxF), and it is possible that linguistic aesthetic preferences have shifted
17 since the 1990s when Crystal conducted his study. Such shifts could further explain
18 differences between our findings and his observations.
19 Irrespective of the ratings across conditions, our study revealed that participants’ ratings were
20 consistent across the two rounds of rating, indicating that within individuals, perceptions of
21 word appeal are stable rather than arbitrary. This aligns with research from other modalities,
22 such as music, where listeners demonstrate a high level of internal stability and consistency
23 in their ratings [(Juslin et al., 2023)](https://www.zotero.org/google-docs/?3kqsC4). The fact that we achieved similar results for linguistic stimuli
24 suggests that a systematic investigation of words’ aesthetic appeal based on ratings is
1 feasible, reflects true aesthetic preferences, and is not merely reliant on spontaneous
2 impulses.

## 3 5.2. Memory and recall

4 Regarding the memorization of words with different levels of appeal, we found that the words
5 from our appealing condition were recalled most often. While this aligns with our initial
6 prediction that words designed to be appealing would be best remembered, it is important to
7 interpret this finding cautiously, as these words did not receive the highest ratings in our rating
8 task (see discussion above). Still, notably, when comparing the appeal ratings of recalled
9 versus non-recalled words – focusing on the actual ratings rather than the conditions – we
10 found that recalled words received significantly higher ratings than those not recalled. This
11 suggests a general link between perceived appeal and word memory, with higher appeal being
12 correlated with higher recall rates.
13 The occurrence frequency of speech sounds and phonotactic patterns may not only influence
14 the ratings of aesthetic appeal (as discussed above) but could also play a significant role in
15 word memory and recall. Typically, familiar patterns – such as those resembling common
16 features of the languages speakers know – are more likely to be remembered [(e.g., Luef &](https://www.zotero.org/google-docs/?9qJQJ7)
17 [Resnik, 2023; Popov & Reder, 2020; Poppenk et al., 2010)](https://www.zotero.org/google-docs/?9qJQJ7). Given the complex relationship
18 between the frequency and appeal of speech sounds (as discussed above) and in turn also
19 between appeal and memory, it would be valuable to disentangle the influence of occurrence
20 frequency in a separate experiment. Such a study could systematically vary exposure
21 frequency to specific (pseudo-)words and patterns to assess how these factors independently
22 influence both appeal and memory.
1 Our findings on the perceived appeal and memorability of words align with research in other
2 domains of aesthetic perception. For example, studies have shown that facial prototypicality
3 enhances attractiveness, with more average or prototypical faces being rated as more
4 appealing than distinctive ones [(e.g., Rhodes & Tremewan, 1996; Trujillo et al., 2014)](https://www.zotero.org/google-docs/?AyydcW).
5 However, [Sarno & Alley (1997)](https://www.zotero.org/google-docs/?9oEWWG) demonstrated that distinctiveness enhances memorability,
6 with less prototypical faces often being easier to recognize. These findings are consistent with
7 other research that postulates that appeal and memorability are driven by different cognitive
8 mechanisms: appeal is related to prototypicality and familiarity [(Reber et al., 2004)](https://www.zotero.org/google-docs/?7F09l2), whereas
9 memorability relies on distinctiveness [(Reed Hunt, 2006)](https://www.zotero.org/google-docs/?nOHiKI). This mirrors the results of our study,
10 where stimuli from the neutral condition were rated as most appealing but were only
11 moderately memorable, while stimuli from the appealing condition were more memorable
12 despite receiving lower overall appeal ratings.

## 13 5.3. Outlook and conclusions

14 Our study offers initial insights into the relationship between the aesthetic appeal of linguistic
15 patterns and its link to memory. However, additional research is necessary to develop a more
16 comprehensive understanding of this connection. For instance, while our investigation focused
17 exclusively on speech sounds, future studies could expand this scope to encompass broader
18 linguistic patterns, such as phonotactic patterns, word stress patterns or intonation contours.
19 Additionally, it is difficult to disentangle aesthetic appeal from related concepts that may
20 influence how we perceive linguistic patterns. For example, factors such as naturalness, which
21 is linked to the frequency of patterns in languages that the speakers are typically exposed to,
22 or complexity, which can manifest in cognitive processing effort, may also play critical roles in
23 shaping perceptions of appeal [(cf. Madison & Schiölde, 2017; McDougall et al., 2016; Van](https://www.zotero.org/google-docs/?rtuRvN)
24 [Geert & Wagemans, 2020 i](https://www.zotero.org/google-docs/?rtuRvN)n the visual and musical domain[)](https://www.zotero.org/google-docs/?6FcAne). Future studies should aim to
1 control or isolate these factors by manipulating the input frequency of artificial stimuli or
2 incorporating phoneme and phonotactic frequencies more systematically into stimulus design.
3 Additionally, experiments could explore the directionality of the causal relationship between
4 appeal and memory to disentangle whether patterns are more memorable because they are
5 perceived as appealing, or whether patterns are perceived as more appealing because they
6 are more memorable.
7 Further studies should also consider including participants of diverse linguistic backgrounds
8 to determine whether the aesthetic perception of speech sounds is driven by cross-linguistic
9 cognitive constraints or shaped by cultural influences [(as, e.g., Anikin et al., 2023 did for whole](https://www.zotero.org/google-docs/?fbHVbu)
10 [languages)](https://www.zotero.org/google-docs/?fbHVbu). Finally, considering the multimodal nature of communication [(e.g.,](https://www.zotero.org/google-docs/?ueky8r) [Holler &](https://www.zotero.org/google-docs/?ueky8r)
11 [Levinson, 2019; Perniss, 2018)](https://www.zotero.org/google-docs/?ueky8r), examining the appeal of gestures or signs might provide a
12 valuable addition to the investigation of spoken and written linguistic patterns.
13 Our findings that aesthetic appeal and word memory are linked, and that appealing patterns

14	may be easier to learn and remember have implications for both practical and theoretical

15 domains. On a practical level, our findings can be used to inspire practices and applications
16 for language teaching and marketing by leveraging the appeal of patterns to acquire
17 languages or promote products more effectively [(e.g.,](https://www.zotero.org/google-docs/?0G0SAD) [Pogacar et al., 2015)](https://www.zotero.org/google-docs/?0G0SAD). On a theoretical
18 level, the learning advantage that appealing patterns may have may facilitate their adoption
19 and transmission within communities as well as across speaker generations [(e.g., Enfield,](https://www.zotero.org/google-docs/?3N6fyx)
20 [2014; Kirby et al., 2014)](https://www.zotero.org/google-docs/?3N6fyx). Over time, these patterns could become embedded in cultural
21 artifacts such as literature, poetry or music, further promoting their persistence and spread.
22 Appeal may therefore serve as a driving force in cultural language evolution, shaping the
23 transmission and transformation of linguistic patterns over time. Thus, our study provides an
24 initial step towards understanding the links between aesthetic appeal, memory, and cultural
25 language evolution, paving the way for larger research avenues.

# 1 6. Data availability statement

2 All data and R files used for the analysis are available in the Open Science Framework
3 repository and can be accessed at
4 <https://osf.io/npe2g/?view_only=f45c8373ecbd4c43abcc4ff4e00eb267>.

5

# 6 7. Funding

7	This work was supported by a XXX grant from XXX (grant number: XXX) awarded to XXX.

# 8 8. References

9 [Aleem, H., Pombo, M., Correa-Herran, I., & Grzywacz, N. M. (2019). Is Beauty in the Eye of](https://www.zotero.org/google-docs/?F5fqEf)
10 [the Beholder or an Objective Truth? A Neuroscientific Answer. In J. L. Contreras-Vidal,](https://www.zotero.org/google-docs/?F5fqEf)
11 [D. Robleto, J. G. Cruz-Garza, J. M. Azorín, & C. S. Nam (Eds.),](https://www.zotero.org/google-docs/?F5fqEf) [*Mobile Brain-Body*](https://www.zotero.org/google-docs/?F5fqEf)
12 [*Imaging and the Neuroscience of Art, Innovation and Creativity*](https://www.zotero.org/google-docs/?F5fqEf) [(Vol. 10, pp. 101–110).](https://www.zotero.org/google-docs/?F5fqEf)
13 [Springer International Publishing. https://doi.org/10.1007/978-3-030-24326-5_11](https://www.zotero.org/google-docs/?F5fqEf)
14 [Anderson, A. K., Wais, P. E., & Gabrieli, J. D. E. (2006). Emotion enhances remembrance of](https://www.zotero.org/google-docs/?r7qMEm)
15 [neutral events past.](https://www.zotero.org/google-docs/?r7qMEm) [*Proceedings of the National Academy of Sciences*,](https://www.zotero.org/google-docs/?r7qMEm) [*103*(5), 1599–](https://www.zotero.org/google-docs/?r7qMEm)
16 [1604. https://doi.org/10.1073/pnas.0506308103](https://www.zotero.org/google-docs/?r7qMEm)
17 [Anikin, A., Aseyev, N., & Erben Johansson, N. (2023). Do some languages sound more](https://www.zotero.org/google-docs/?r7qMEm)
18 [beautiful than others?](https://www.zotero.org/google-docs/?r7qMEm) [*Proceedings of the National Academy of Sciences*,](https://www.zotero.org/google-docs/?r7qMEm) [*120*(17),](https://www.zotero.org/google-docs/?r7qMEm)
19 [e2218367120. https://doi.org/10.1073/pnas.2218367120](https://www.zotero.org/google-docs/?r7qMEm)
20 A"dacitQ ľcam. (2022). *€udaciťQ: Iíee audio ediťoí a⭲d íecoídeí* (Vcísio⭲ «.2.1) [Comp"tcí
21 sortwaíc]. Rctíic:cd ríom [kttps://www.a"dacitQtcam.oíg](https://www.audacityteam.org/)
22 [Baayen, R. H. (2008).](https://www.zotero.org/google-docs/?F5fqEf) [*Analyzing Linguistic Data: A Practical Introduction to Statistics using R*](https://www.zotero.org/google-docs/?F5fqEf)
23 [(1st ed.). Cambridge University Press. https://doi.org/10.1017/CBO9780511801686](https://www.zotero.org/google-docs/?F5fqEf)
24 [Barr, D. J. (2013). Random effects structure for testing interactions in linear mixed-effects](https://www.zotero.org/google-docs/?F5fqEf)
25 [models.](https://www.zotero.org/google-docs/?F5fqEf) [*Frontiers in Psychology*,](https://www.zotero.org/google-docs/?F5fqEf) [*4*. https://doi.org/10.3389/fpsyg.2013.00328](https://www.zotero.org/google-docs/?F5fqEf)
26 Baítoń, K. (2024). *MuMI⭲: Mulťi-½odel i⭲reíe⭲ce* (Vcísio⭲ 1.4®.4) [R packagc ma⭲"al].
   1 Rctíic:cd ríom [kttps://cía⭲.í-píojcct.oíg/packagc=M"MI⭲](https://cran.r-project.org/package%3DMuMIn)
   2 [Bates, D., Mächler, M., Bolker, B., & Walker, S. (2015). Fitting Linear Mixed-Effects Models](https://www.zotero.org/google-docs/?F5fqEf)
   3 [Using lme4.](https://www.zotero.org/google-docs/?F5fqEf) [*Journal of Statistical Software*,](https://www.zotero.org/google-docs/?F5fqEf) [*67*(1). https://doi.org/10.18637/jss.v067.i01](https://www.zotero.org/google-docs/?F5fqEf)
   4 [Baumeister, R. F., Bratslavsky, E., Finkenauer, C., & Vohs, K. D. (2001). Bad is Stronger](https://www.zotero.org/google-docs/?r7qMEm)
   5 [than Good.](https://www.zotero.org/google-docs/?r7qMEm) [*Review of General Psychology*,](https://www.zotero.org/google-docs/?r7qMEm) [*5*(4), 323–370.](https://www.zotero.org/google-docs/?r7qMEm) [https://doi.org/10.1037/1089-](https://doi.org/10.1037/1089-2680.5.4.323)
   6 [2680.5.4.323](https://doi.org/10.1037/1089-2680.5.4.323)
   7 [Berlyne, D. E. (1970). Novelty, complexity, and hedonic value.](https://www.zotero.org/google-docs/?SDqa6m) [*Perception & Psychophysics*,](https://www.zotero.org/google-docs/?SDqa6m)

8	[*8*(5), 279–286.](https://www.zotero.org/google-docs/?SDqa6m) [https://doi.org/10.3758/BF0321259](https://doi.org/10.3758/BF03212593)3

9 [Blondé, P., Sperduti, M., Makowski, D., & Piolino, P. (2022). Bored, distracted, and forgetful:](https://www.zotero.org/google-docs/?r7qMEm)
10 [The impact of mind wandering and boredom on memory encoding.](https://www.zotero.org/google-docs/?r7qMEm) [*Quarterly Journal of*](https://www.zotero.org/google-docs/?r7qMEm)
11 [*Experimental Psychology*,](https://www.zotero.org/google-docs/?r7qMEm) [*75*(1), 53–69. https://doi.org/10.1177/17470218211026301](https://www.zotero.org/google-docs/?r7qMEm)
12 [Blood, A. J., & Zatorre, R. J. (2001). Intensely pleasurable responses to music correlate with](https://www.zotero.org/google-docs/?r7qMEm)
13 [activity in brain regions implicated in reward and emotion.](https://www.zotero.org/google-docs/?r7qMEm) [*Proceedings of the National*](https://www.zotero.org/google-docs/?r7qMEm)
14 [*Academy of Sciences*,](https://www.zotero.org/google-docs/?r7qMEm) [*98*(20), 11818–11823. https://doi.org/10.1073/pnas.191355898](https://www.zotero.org/google-docs/?r7qMEm)
15 Ckíistc⭲sc⭲, R. H. B., & Bíockkorr, P. (201«). A⭲alQsis or sc⭲soíQ íati⭲gs data witk
16 c"m"lati:c li⭲k modcls. *Jouí⭲al de La Sociéťé Iía⭲çaise de Sťaťisťique ł Re»ue de*
17 *Sťaťisťique €ppliquée*, 154(«), 5®–79.
18 Ckíistc⭲sc⭲, R. H. B. (201®). *Cu½ulaťi»e li⭲k ½odels roí oídi⭲al íegíessio⭲**iťh ťhe R package*
19 *oídi⭲al* [R packagc ma⭲"al]. Rctíic:cd ríom [kttps://cía⭲.í-píojcct.oíg/packagc=oídi⭲al](https://cran.r-project.org/package%3Dordinal)
20 Ckíistc⭲sc⭲, R. H. B. (2019). *oídi⭲al - Regíessio⭲ Models roí Oídi⭲al Daťa* [R packagc ma⭲"al].
21 Rctíic:cd ríom [kttps://cía⭲.í-píojcct.oíg/packagc=oídi⭲al](https://cran.r-project.org/package%3Dordinal)
22 Christensen, R. H. B. (2023). *ordinal: Regression models for ordinal data* (Version 2023.12-
23 4) [R package manual]. Retrieved from [https://cran.r-project.org/package=ordina](https://cran.r-project.org/package%3Dordinal)l
24 [Cohen, W. W., Ravikumar, P., & Fienberg, S. E. (2003). A Comparison of String Distance](https://www.zotero.org/google-docs/?F5fqEf)
25 [Metrics for Name-Matching Tasks.](https://www.zotero.org/google-docs/?F5fqEf) [*IIWeb*.](https://www.zotero.org/google-docs/?F5fqEf)
26 [https://api.semanticscholar.org/CorpusID:10625463](https://www.zotero.org/google-docs/?F5fqEf)
27 [Crystal, D. (1995). Phonaesthetically speaking.](https://www.zotero.org/google-docs/?r7qMEm) [*English Today*,](https://www.zotero.org/google-docs/?r7qMEm) [*11*(2), 8–12.](https://www.zotero.org/google-docs/?r7qMEm)

28	<https://doi.org/10.1017/S026607840000818X>

29 [Cumming, G., & Finch, S. (2005). Inference by Eye: Confidence Intervals and How to Read](https://www.zotero.org/google-docs/?DWxvrG)
30 [Pictures of Data.](https://www.zotero.org/google-docs/?DWxvrG) [*American Psychologist*,](https://www.zotero.org/google-docs/?DWxvrG) [*60*(2), 170–180.](https://www.zotero.org/google-docs/?DWxvrG) [https://doi.org/10.1037/0003-](https://doi.org/10.1037/0003-066X.60.2.170)
31 [066X.60.2.170](https://doi.org/10.1037/0003-066X.60.2.170)
32 [Dewey, J. (2005).](https://www.zotero.org/google-docs/?r7qMEm) [*Art as experience*. Berkeley Publishing Group.](https://www.zotero.org/google-docs/?r7qMEm)
33 Dobso⭲, A. J. (2002). *€⭲ i⭲ťíoducťio⭲ ťo ge⭲eíalized li⭲eaí ½odels*. Boca Rato⭲, FL: Ckapma⭲
34 & Hall.
   1 [Enfield, N. J. (2014). Transmission biases in the cultural evolution of language: Towards an](https://www.zotero.org/google-docs/?SDqa6m)
   2 [explanatory framework. In D. Dor, C. Knight, & J. Lewis (Eds.),](https://www.zotero.org/google-docs/?SDqa6m) [*The Social Origins of*](https://www.zotero.org/google-docs/?SDqa6m)
   3 [*Language*](https://www.zotero.org/google-docs/?SDqa6m) [(pp. 325–335). Oxford University Press.](https://www.zotero.org/google-docs/?SDqa6m)
   4 [https://doi.org/10.1093/acprof:oso/9780199665327.003.0023](https://www.zotero.org/google-docs/?SDqa6m)
   5 [Hayden, R. E. (1950). The Relative Frequency of Phonemes in General-American English.](https://www.zotero.org/google-docs/?SDqa6m)

6	[WORD,](https://www.zotero.org/google-docs/?SDqa6m) [*6*(3), 217–223. https://doi.org/10.1080/00437956.1950.11659381](https://www.zotero.org/google-docs/?SDqa6m)

7	[Hayden, Rebecca E. (1950). “The Relative Frequency of Phonemes in General-American](https://www.zotero.org/google-docs/?GDIIIF)

8	[English.” WORD 6(3):217–23. doi: 10.1080/00437956.1950.11659381.](https://www.zotero.org/google-docs/?GDIIIF)

9 [Holler, J., & Levinson, S. C. (2019). Multimodal Language Processing in Human](https://www.zotero.org/google-docs/?SDqa6m)
10 [Communication.](https://www.zotero.org/google-docs/?SDqa6m) [*Trends in Cognitive Sciences*,](https://www.zotero.org/google-docs/?SDqa6m) [*23*(8), 639–652.](https://www.zotero.org/google-docs/?SDqa6m)
11 [https://doi.org/10.1016/j.tics.2019.05.006](https://www.zotero.org/google-docs/?SDqa6m)
12 [Isik, A. I., & Vessel, E. A. (2021). From Visual Perception to Aesthetic Appeal: Brain](https://www.zotero.org/google-docs/?r7qMEm)
13 [Responses to Aesthetically Appealing Natural Landscape Movies.](https://www.zotero.org/google-docs/?r7qMEm) [*Frontiers in Human*](https://www.zotero.org/google-docs/?r7qMEm)
14 [*Neuroscience*,](https://www.zotero.org/google-docs/?r7qMEm) [*15*, 676032. https://doi.org/10.3389/fnhum.2021.676032](https://www.zotero.org/google-docs/?r7qMEm)
15 [Jacobsen, T., & Beudt, S. (2017). Stability and Variability in Aesthetic Experience: A Review.](https://www.zotero.org/google-docs/?SDqa6m)
16 [*Frontiers in Psychology*,](https://www.zotero.org/google-docs/?SDqa6m) [*8*. https://doi.org/10.3389/fpsyg.2017.00143](https://www.zotero.org/google-docs/?SDqa6m)
17 [Jaro, M. A. (1989). Advances in Record-Linkage Methodology as Applied to Matching the](https://www.zotero.org/google-docs/?F5fqEf)
18 [1985 Census of Tampa, Florida.](https://www.zotero.org/google-docs/?F5fqEf) [*Journal of the American Statistical Association*,](https://www.zotero.org/google-docs/?F5fqEf)
19 [*84*(406), 414–420. https://doi.org/10.1080/01621459.1989.10478785](https://www.zotero.org/google-docs/?F5fqEf)
20 [Jespersen, O. (2013).](https://www.zotero.org/google-docs/?r7qMEm) [*Language*](https://www.zotero.org/google-docs/?r7qMEm) [(0 ed.). Routledge. https://doi.org/10.4324/9780203715895](https://www.zotero.org/google-docs/?r7qMEm)
21 [Joe, H. (2008). Accuracy of Laplace approximation for discrete response mixed models.](https://www.zotero.org/google-docs/?F5fqEf)
22 [*Computational Statistics & Data Analysis*,](https://www.zotero.org/google-docs/?F5fqEf) [*52*(12), 5066–5074.](https://www.zotero.org/google-docs/?F5fqEf)
23 [https://doi.org/10.1016/j.csda.2008.05.002](https://www.zotero.org/google-docs/?F5fqEf)
24 [Juslin, P. N., Ingmar, E., & Danielsson, J. (2023). Aesthetic judgments of music: Reliability,](https://www.zotero.org/google-docs/?SDqa6m)
25 [consistency, criteria, self-insight, and expertise.](https://www.zotero.org/google-docs/?SDqa6m) [*Psychology of Aesthetics, Creativity,*](https://www.zotero.org/google-docs/?SDqa6m)
26 [*and the Arts*,](https://www.zotero.org/google-docs/?SDqa6m) [*17*(2), 193–210. https://doi.org/10.1037/aca0000403](https://www.zotero.org/google-docs/?SDqa6m)
27 [Kensinger, E. A., & Corkin, S. (2003). Memory enhancement for emotional words: Are](https://www.zotero.org/google-docs/?r7qMEm)
28 [emotional words more vividly remembered than neutral words?](https://www.zotero.org/google-docs/?r7qMEm) [*Memory & Cognition*,](https://www.zotero.org/google-docs/?r7qMEm)
29 [*31*(8), 1169–1180. https://doi.org/10.3758/BF03195800](https://www.zotero.org/google-docs/?r7qMEm)
30 [Kirby, S., Griffiths, T., & Smith, K. (2014). Iterated learning and the evolution of language.](https://www.zotero.org/google-docs/?SDqa6m)
31 [*Current Opinion in Neurobiology*,](https://www.zotero.org/google-docs/?SDqa6m) [*28*, 108–114.](https://www.zotero.org/google-docs/?SDqa6m)
32 [https://doi.org/10.1016/j.conb.2014.07.014](https://www.zotero.org/google-docs/?SDqa6m)
33 [Klink, R. R., & Athaide, G. A. (2012). Creating brand personality with brand names.](https://www.zotero.org/google-docs/?r7qMEm)

34	[*Marketing Letters*,](https://www.zotero.org/google-docs/?r7qMEm) [*23*(1), 109–117. https://doi.org/10.1007/s11002-011-9140-7](https://www.zotero.org/google-docs/?r7qMEm)

1 [Kogan, V. V., & Reiterer, S. M. (2021). Eros, Beauty, and Phon-Aesthetic Judgements of](https://www.zotero.org/google-docs/?r7qMEm)
2 [Language Sound. We Like It Flat and Fast, but Not Melodious. Comparing Phonetic and](https://www.zotero.org/google-docs/?r7qMEm)
3 [Acoustic Features of 16 European Languages.](https://www.zotero.org/google-docs/?r7qMEm) [*Frontiers in Human Neuroscience*,](https://www.zotero.org/google-docs/?r7qMEm) [*15*,](https://www.zotero.org/google-docs/?r7qMEm)
4 [578594. https://doi.org/10.3389/fnhum.2021.578594](https://www.zotero.org/google-docs/?r7qMEm)
5 [Körner, A., & Rummer, R. (2023). Valence sound symbolism across language families: A](https://www.zotero.org/google-docs/?r7qMEm)
6 [comparison between Japanese and German.](https://www.zotero.org/google-docs/?r7qMEm) [*Language and Cognition*,](https://www.zotero.org/google-docs/?r7qMEm) [*15*(2), 337–354.](https://www.zotero.org/google-docs/?r7qMEm)
7 [https://doi.org/10.1017/langcog.2022.39](https://www.zotero.org/google-docs/?r7qMEm)
8 [Kousta, S.-T., Vinson, D. P., & Vigliocco, G. (2009). Emotion words, regardless of polarity,](https://www.zotero.org/google-docs/?r7qMEm)
9 [have a processing advantage over neutral words.](https://www.zotero.org/google-docs/?r7qMEm) [*Cognition*,](https://www.zotero.org/google-docs/?r7qMEm) [*112*(3), 473–481.](https://www.zotero.org/google-docs/?r7qMEm)
10 [https://doi.org/10.1016/j.cognition.2009.06.007](https://www.zotero.org/google-docs/?r7qMEm)
11 [Lev-Ari, S., & McKay, R. (2023). The sound of swearing: Are there universal patterns in](https://www.zotero.org/google-docs/?r7qMEm)
12 [profanity?](https://www.zotero.org/google-docs/?r7qMEm) [*Psychonomic Bulletin & Review*,](https://www.zotero.org/google-docs/?r7qMEm) [*30*(3), 1103–1114.](https://www.zotero.org/google-docs/?r7qMEm)
13 [https://doi.org/10.3758/s13423-022-02202-0](https://www.zotero.org/google-docs/?r7qMEm)
14 [Luef, E. M., & Resnik, P. (2023). Phonotactic Probabilities and Sub-syllabic Segmentation in](https://www.zotero.org/google-docs/?SDqa6m)
15 [Language Learning.](https://www.zotero.org/google-docs/?SDqa6m) [*Theory and Practice of Second Language Acquisition*,](https://www.zotero.org/google-docs/?SDqa6m) [*9*(1), 1–31.](https://www.zotero.org/google-docs/?SDqa6m)
16 [https://doi.org/10.31261/TAPSLA.12468](https://www.zotero.org/google-docs/?SDqa6m)
17 [Luef, Eva Maria, and Pia Resnik. (2023). “Phonotactic Probabilities and Sub-Syllabic](https://www.zotero.org/google-docs/?GDIIIF)
18 [Segmentation in Language Learning.”](https://www.zotero.org/google-docs/?GDIIIF) [*Theory and Practice of Second Language*](https://www.zotero.org/google-docs/?GDIIIF)
19 [*Acquisition*](https://www.zotero.org/google-docs/?GDIIIF) [9(1):1–31. doi: 10.31261/TAPSLA.12468.](https://www.zotero.org/google-docs/?GDIIIF)
20 [Madison, G., & Schiölde, G. (2017). Repeated Listening Increases the Liking for Music](https://www.zotero.org/google-docs/?SDqa6m)
21 [Regardless of Its Complexity: Implications for the Appreciation and Aesthetics of Music.](https://www.zotero.org/google-docs/?SDqa6m)
22 [*Frontiers in Neuroscience*,](https://www.zotero.org/google-docs/?SDqa6m) [*11*. https://doi.org/10.3389/fnins.2017.00147](https://www.zotero.org/google-docs/?SDqa6m)
23 [Matzinger, T., Specker, E., Ritt, N., & Fitch, T. (2021). Aesthetic perception of prosodic](https://www.zotero.org/google-docs/?r7qMEm)
24 [patterns as a factor in speech segmentation.](https://www.zotero.org/google-docs/?r7qMEm) [*Proceedings of the Annual Meeting of the*](https://www.zotero.org/google-docs/?r7qMEm)
25 [*Cognitive Science Society*,](https://www.zotero.org/google-docs/?r7qMEm) [*43*(43). https://escholarship.org/uc/item/4tm94762](https://www.zotero.org/google-docs/?r7qMEm)
26 [McCullagh, P., & Nelder, J. A. (1989).](https://www.zotero.org/google-docs/?F5fqEf) [*Generalized Linear Models*. Springer US.](https://www.zotero.org/google-docs/?F5fqEf)

27	[https://doi.org/10.1007/978-1-4899-3242-6](https://www.zotero.org/google-docs/?F5fqEf)

28 [McDougall, S., Reppa, I., Kulik, J., & Taylor, A. (2016). What makes icons appealing? The](https://www.zotero.org/google-docs/?SDqa6m)
29 [role of processing fluency in predicting icon appeal in different task contexts.](https://www.zotero.org/google-docs/?SDqa6m) [*Applied*](https://www.zotero.org/google-docs/?SDqa6m)
30 [*Ergonomics*,](https://www.zotero.org/google-docs/?SDqa6m) [*55*, 156–172. https://doi.org/10.1016/j.apergo.2016.02.006](https://www.zotero.org/google-docs/?SDqa6m)
31 [McDougall, Siné, Irene Reppa, Jozef Kulik, and Alisdair Taylor. (2016). “What Makes Icons](https://www.zotero.org/google-docs/?GDIIIF)
32 [Appealing? The Role of Processing Fluency in Predicting Icon Appeal in Different Task](https://www.zotero.org/google-docs/?GDIIIF)
33 [Contexts.”](https://www.zotero.org/google-docs/?GDIIIF) [*Applied Ergonomics*](https://www.zotero.org/google-docs/?GDIIIF) [55:156–72. doi: 10.1016/j.apergo.2016.02.006.](https://www.zotero.org/google-docs/?GDIIIF)
34 [Mines, M. A., Hanson, B. F., & Shoup, J. E. (1978). Frequency of Occurrence of Phonemes](https://www.zotero.org/google-docs/?SDqa6m)
   1 [in Conversational English.](https://www.zotero.org/google-docs/?SDqa6m) [*Language and Speech*,](https://www.zotero.org/google-docs/?SDqa6m) [*21*(3), 221–241.](https://www.zotero.org/google-docs/?SDqa6m)
   2 [https://doi.org/10.1177/002383097802100302](https://www.zotero.org/google-docs/?SDqa6m)
   3 [Mooshammer, C., Bobeck, D., Hornecker, H., Meinhardt, K., Olina, O., Walch, M. C., & Xia,](https://www.zotero.org/google-docs/?r7qMEm)
   4 [Q. (2023). Does Orkish Sound Evil? Perception of Fantasy Languages and Their](https://www.zotero.org/google-docs/?r7qMEm)
   5 [Phonetic and Phonological Characteristics.](https://www.zotero.org/google-docs/?r7qMEm) [*Language and Speech*,](https://www.zotero.org/google-docs/?r7qMEm)
   6 [00238309231202944. https://doi.org/10.1177/00238309231202944](https://www.zotero.org/google-docs/?r7qMEm)
   7 [Nakagawa, S., & Schielzeth, H. (2013). A general and simple method for obtaining](https://www.zotero.org/google-docs/?F5fqEf) [*R*^2^](https://www.zotero.org/google-docs/?F5fqEf) [from](https://www.zotero.org/google-docs/?F5fqEf)
   8 [generalized linear mixed‐ effects models.](https://www.zotero.org/google-docs/?F5fqEf) [*Methods in Ecology and Evolution*,](https://www.zotero.org/google-docs/?F5fqEf) [*4*(2), 133–](https://www.zotero.org/google-docs/?F5fqEf)
   9 [142. https://doi.org/10.1111/j.2041-210x.2012.00261.x](https://www.zotero.org/google-docs/?F5fqEf)
   10 [Payne, J. D., & Kensinger, E. A. (2018). Stress, sleep, and the selective consolidation of](https://www.zotero.org/google-docs/?r7qMEm)
   11 [emotional memories.](https://www.zotero.org/google-docs/?r7qMEm) [*Current Opinion in Behavioral Sciences*,](https://www.zotero.org/google-docs/?r7qMEm) [*19*, 36–43.](https://www.zotero.org/google-docs/?r7qMEm)
   12 [https://doi.org/10.1016/j.cobeha.2017.09.006](https://www.zotero.org/google-docs/?r7qMEm)
   13 [Perniss, P. (2018). Why We Should Study Multimodal Language.](https://www.zotero.org/google-docs/?SDqa6m) [*Frontiers in Psychology*,](https://www.zotero.org/google-docs/?SDqa6m) [*9*,](https://www.zotero.org/google-docs/?SDqa6m)

14	[1109. https://doi.org/10.3389/fpsyg.2018.01109](https://www.zotero.org/google-docs/?SDqa6m)

15 [Pinheiro, J. C., & Bates, D. M. (1995). Approximations to the Log-Likelihood Function in the](https://www.zotero.org/google-docs/?F5fqEf)
16 [Nonlinear Mixed-Effects Model.](https://www.zotero.org/google-docs/?F5fqEf) [*Journal of Computational and Graphical Statistics*,](https://www.zotero.org/google-docs/?F5fqEf) [*4*(1),](https://www.zotero.org/google-docs/?F5fqEf)
17 [12–35. https://doi.org/10.1080/10618600.1995.10474663](https://www.zotero.org/google-docs/?F5fqEf)
18 [Pogacar, R., Plant, E., Rosulek, L. F., & Kouril, M. (2015). Sounds good: Phonetic sound](https://www.zotero.org/google-docs/?r7qMEm)
19 [patterns in top brand names.](https://www.zotero.org/google-docs/?r7qMEm) [*Marketing Letters*,](https://www.zotero.org/google-docs/?r7qMEm) [*26*(4), 549–563.](https://www.zotero.org/google-docs/?r7qMEm)
20 [https://doi.org/10.1007/s11002-014-9288-z](https://www.zotero.org/google-docs/?r7qMEm)
21 [Popov, V., & Reder, L. M. (2020). Frequency effects on memory: A resource-limited theory.](https://www.zotero.org/google-docs/?SDqa6m)
22 [*Psychological Review*,](https://www.zotero.org/google-docs/?SDqa6m) [*127*(1), 1–46. https://doi.org/10.1037/rev0000161](https://www.zotero.org/google-docs/?SDqa6m)
23 [Poppenk, J., Köhler, S., & Moscovitch, M. (2010). Revisiting the novelty effect: When](https://www.zotero.org/google-docs/?SDqa6m)
24 [familiarity, not novelty, enhances memory.](https://www.zotero.org/google-docs/?SDqa6m) [*Journal of Experimental Psychology:*](https://www.zotero.org/google-docs/?SDqa6m)
25 [*Learning, Memory, and Cognition*,](https://www.zotero.org/google-docs/?SDqa6m) [*36*(5), 1321–1330. https://doi.org/10.1037/a0019900](https://www.zotero.org/google-docs/?SDqa6m)
26 Píoliric. (2014). *Níoliric* [Comp"tcí sortwaíc] (Vcísio⭲ Ja⭲"aíQ 2025). Lo⭲do⭲, UK. CopQíigkt
27 2024. Rctíic:cd ríom [kttps://www.píoliric.com](https://www.prolific.com/)
28 R Coíc ľcam. (202«). *R: € la⭲guage a⭲d e⭲»iío⭲½e⭲ť roí sťaťisťical co½puťi⭲g* [Comp"tcí
29 sortwaíc]. Vic⭲⭲a, A"stíia: R Fo"⭲datio⭲ roí Statistical Comp"ti⭲g. Rctíic:cd Ja⭲"aíQ 9,
30 2025, ríom [kttps://www.R-píojcct.oí](https://www.r-project.org/)g
31 [Reber, R., Schwarz, N., & Winkielman, P. (2004). Processing Fluency and Aesthetic](https://www.zotero.org/google-docs/?SDqa6m)
32 [Pleasure: Is Beauty in the Perceiver’s Processing Experience?](https://www.zotero.org/google-docs/?SDqa6m) [*Personality and Social*](https://www.zotero.org/google-docs/?SDqa6m)
33 [*Psychology Review*,](https://www.zotero.org/google-docs/?SDqa6m) [*8*(4), 364–382. https://doi.org/10.1207/s15327957pspr0804_3](https://www.zotero.org/google-docs/?SDqa6m)
34 [Reed Hunt, R. (2006). The Concept of Distinctiveness in Memory Research. In R. R. Hunt &](https://www.zotero.org/google-docs/?SDqa6m)
   1 [J. B. Worthen (Eds.),](https://www.zotero.org/google-docs/?SDqa6m) [*Distinctiveness and Memory*](https://www.zotero.org/google-docs/?SDqa6m) [(pp. 2–25). Oxford University Press.](https://www.zotero.org/google-docs/?SDqa6m)
   2 [https://doi.org/10.1093/acprof:oso/9780195169669.003.0001](https://www.zotero.org/google-docs/?SDqa6m)
   3 [Reppa, I., & McDougall, S. (2015). When the going gets tough the beautiful get going:](https://www.zotero.org/google-docs/?r7qMEm)
   4 [Aesthetic appeal facilitates task performance.](https://www.zotero.org/google-docs/?r7qMEm) [*Psychonomic Bulletin & Review*,](https://www.zotero.org/google-docs/?r7qMEm) [*22*(5),](https://www.zotero.org/google-docs/?r7qMEm)
   5 [1243–1254. https://doi.org/10.3758/s13423-014-0794-z](https://www.zotero.org/google-docs/?r7qMEm)
   6 [Rhodes, G., & Tremewan, T. (1996). Averageness, Exaggeration, and Facial Attractiveness.](https://www.zotero.org/google-docs/?SDqa6m)
   7 [*Psychological Science*,](https://www.zotero.org/google-docs/?SDqa6m) [*7*(2), 105–110. https://doi.org/10.1111/j.1467-](https://www.zotero.org/google-docs/?SDqa6m)
   8 [9280.1996.tb00338.x](https://www.zotero.org/google-docs/?SDqa6m)
   9 [Rozin, P., & Royzman, E. B. (2001). Negativity Bias, Negativity Dominance, and Contagion.](https://www.zotero.org/google-docs/?r7qMEm)
   10 [*Personality and Social Psychology Review*,](https://www.zotero.org/google-docs/?r7qMEm) [*5*(4), 296–320.](https://www.zotero.org/google-docs/?r7qMEm)
   11 [https://doi.org/10.1207/S15327957PSPR0504_2](https://www.zotero.org/google-docs/?r7qMEm)
   12 [Saito, Y. (2007).](https://www.zotero.org/google-docs/?r7qMEm) [*Everyday Aesthetics*](https://www.zotero.org/google-docs/?r7qMEm) [(Vol. 25, Issue 1, pp. 87–95). Oxford University Press.](https://www.zotero.org/google-docs/?r7qMEm)
   13 [Sarno, J. A., & Alley, T. R. (1997). Attractiveness and the Memorability of Faces: Only a](https://www.zotero.org/google-docs/?SDqa6m)
   14 [Matter of Distinctiveness?](https://www.zotero.org/google-docs/?SDqa6m) [*The American Journal of Psychology*,](https://www.zotero.org/google-docs/?SDqa6m) [*110*(1), 81.](https://www.zotero.org/google-docs/?SDqa6m)
   15 [https://doi.org/10.2307/1423702](https://www.zotero.org/google-docs/?SDqa6m)
   16 [Schindler, I., Hosoya, G., Menninghaus, W., Beermann, U., Wagner, V., Eid, M., & Scherer,](https://www.zotero.org/google-docs/?r7qMEm)
   17 [K. R. (2017). Measuring aesthetic emotions: A review of the literature and a new](https://www.zotero.org/google-docs/?r7qMEm)
   18 [assessment tool.](https://www.zotero.org/google-docs/?r7qMEm) [*PLOS ONE*,](https://www.zotero.org/google-docs/?r7qMEm) [*12*(6), e0178899.](https://www.zotero.org/google-docs/?r7qMEm)
   19 [https://doi.org/10.1371/journal.pone.0178899](https://www.zotero.org/google-docs/?r7qMEm)
   20 [Sharot, T., Delgado, M. R., & Phelps, E. A. (2004). How emotion enhances the feeling of](https://www.zotero.org/google-docs/?r7qMEm)
   21 [remembering.](https://www.zotero.org/google-docs/?r7qMEm) [*Nature Neuroscience*,](https://www.zotero.org/google-docs/?r7qMEm) [*7*(12), 1376–1380. https://doi.org/10.1038/nn1353](https://www.zotero.org/google-docs/?r7qMEm)
   22 [Sluckin, W., Colman, A. M., & Hargreaves, D. J. (1980). Liking words as a function of the](https://www.zotero.org/google-docs/?SDqa6m)
   23 [experienced frequency of their occurrence.](https://www.zotero.org/google-docs/?SDqa6m) [*British Journal of Psychology*,](https://www.zotero.org/google-docs/?SDqa6m) [*71*(1), 163–](https://www.zotero.org/google-docs/?SDqa6m)
   24 [169. https://doi.org/10.1111/j.2044-8295.1980.tb02742.x](https://www.zotero.org/google-docs/?SDqa6m)
   25 [Tamir, M., & Robinson, M. D. (2007). The Happy Spotlight: Positive Mood and Selective](https://www.zotero.org/google-docs/?r7qMEm)
   26 [Attention to Rewarding Information.](https://www.zotero.org/google-docs/?r7qMEm) [*Personality and Social Psychology Bulletin*,](https://www.zotero.org/google-docs/?r7qMEm) [*33*(8),](https://www.zotero.org/google-docs/?r7qMEm)
   27 [1124–1136. https://doi.org/10.1177/0146167207301030](https://www.zotero.org/google-docs/?r7qMEm)
   28 [Trujillo, L. T., Jankowitsch, J. M., & Langlois, J. H. (2014). Beauty is in the ease of the](https://www.zotero.org/google-docs/?SDqa6m)
   29 [beholding: A neurophysiological test of the averageness theory of facial attractiveness.](https://www.zotero.org/google-docs/?SDqa6m)
   30 [*Cognitive, Affective, & Behavioral Neuroscience*,](https://www.zotero.org/google-docs/?SDqa6m) [*14*(3), 1061–1076.](https://www.zotero.org/google-docs/?SDqa6m)
   31 [https://doi.org/10.3758/s13415-013-0230-2](https://www.zotero.org/google-docs/?SDqa6m)
   32 [Van Geert, E., & Wagemans, J. (2020). Order, complexity, and aesthetic appreciation.](https://www.zotero.org/google-docs/?SDqa6m)
   33 [*Psychology of Aesthetics, Creativity, and the Arts*,](https://www.zotero.org/google-docs/?SDqa6m) [*14*(2), 135–154.](https://www.zotero.org/google-docs/?SDqa6m)
   34 [https://doi.org/10.1037/aca0000224](https://www.zotero.org/google-docs/?SDqa6m)

1	[Vartanian, O., & Goel, V. (2004). Neuroanatomical correlates of aesthetic preference for](https://www.zotero.org/google-docs/?r7qMEm)

2	[paintings:](https://www.zotero.org/google-docs/?r7qMEm) [*NeuroReport*,](https://www.zotero.org/google-docs/?r7qMEm) [*15*(5), 893–897. https://doi.org/10.1097/00001756-200404090-](https://www.zotero.org/google-docs/?r7qMEm)

3	[00032](https://www.zotero.org/google-docs/?r7qMEm)

4 [Vartanian, O., & Nadal, M. (2022).](https://www.zotero.org/google-docs/?r7qMEm) [*The Oxford handbook of empirical aesthetics*. Oxford](https://www.zotero.org/google-docs/?r7qMEm)
5 [University Press.](https://www.zotero.org/google-docs/?r7qMEm)
6 [Vashi, N. A., & Quay, E. R. (2015). Subjective Aspects of Beauty. In N. A. Vashi (Ed.),](https://www.zotero.org/google-docs/?F5fqEf)
7 [*Beauty and Body Dysmorphic Disorder*](https://www.zotero.org/google-docs/?F5fqEf) [(pp. 63–81). Springer International Publishing.](https://www.zotero.org/google-docs/?F5fqEf)
8 [https://doi.org/10.1007/978-3-319-17867-7_4](https://www.zotero.org/google-docs/?F5fqEf)
9 [Winkler, A., Kogan, V. V., & Reiterer, S. M. (2023). Phonaesthetics and personality—Why](https://www.zotero.org/google-docs/?r7qMEm)
10 [we do not only prefer Romance languages.](https://www.zotero.org/google-docs/?r7qMEm) [*Frontiers in Language Sciences*,](https://www.zotero.org/google-docs/?r7qMEm) [*2*,](https://www.zotero.org/google-docs/?r7qMEm)
11 [1043619. https://doi.org/10.3389/flang.2023.1043619](https://www.zotero.org/google-docs/?r7qMEm)
12 [Winkler, W. E. (1990).](https://www.zotero.org/google-docs/?F5fqEf) [*String Comparator Metrics and Enhanced Decision Rules in the*](https://www.zotero.org/google-docs/?F5fqEf)
13 [*Fellegi-Sunter Model of Record Linkage.*](https://www.zotero.org/google-docs/?F5fqEf)
14 https://api.semanticscholar.org/CorpusID:54580585
